# The Mental Health–Acute Coronary Syndrome Continuum: Bidirectional Pathophysiological Links and Clinical Implications

**DOI:** 10.3390/medsci14010138

**Published:** 2026-03-16

**Authors:** Alexandra Herlaș-Pop, Andrei-Flavius Radu, Ada Radu, Gabriela S. Bungau, Delia Mirela Tit, Elena Emilia Babes, Cristiana Bustea

**Affiliations:** 1 Doctoral School of Biological and Biomedical Sciences, University of Oradea, 410087 Oradea, Romania; pop.alexandra@student.uoradea.ro (A.H.-P.); gbungau@uoradea.ro (G.S.B.); dtit@uoradea.ro (D.M.T.); eebabes@uoradea.ro (E.E.B.); cbustea@uoradea.ro (C.B.); 2 Department of Psycho-Neuroscience and Recovery, Faculty of Medicine and Pharmacy, University of Oradea, 410073 Oradea, Romania; 3 Department of Pharmacy, Faculty of Medicine and Pharmacy, University of Oradea, 410028 Oradea, Romania; 4 Department of Medical Disciplines, Faculty of Medicine and Pharmacy, University of Oradea, 410073 Oradea, Romania; 5 Department of Preclinical Disciplines, Faculty of Medicine and Pharmacy, University of Oradea, 410073 Oradea, Romania

**Keywords:** acute coronary syndrome, mental health disorders, depression, bidirectional relationship, anxiety, bipolar disorder, schizophrenia, PTSD, multidisciplinarity

## Abstract

Mental health disorders (MHDs) and acute coronary syndromes (ACSs) demonstrate reciprocal pathophysiological connections with substantial prognostic implications. Despite robust evidence linking MHDs to adverse cardiovascular outcomes, the bidirectional relationship remains inadequately characterized in clinical practice, with limited integration of mental health screening into routine cardiac care pathways. The present narrative review comprehensively presents contemporary data on epidemiology, shared biological mechanisms, clinical consequences, and integrated management strategies across the MHD–ACS continuum. A synthesis of peer-reviewed literature, meta-analyses, observational cohorts, randomized trials, and international guideline documents was performed, focusing on depression, anxiety, post-traumatic stress disorder, bipolar disorder, schizophrenia, and suicidality in relation to ACSs. MHDs are highly prevalent in ACS populations and independently predict increased mortality, major adverse cardiac events, and poorer functional recovery. Shared mechanisms include chronic low-grade inflammation, autonomic imbalance, hypothalamic–pituitary–adrenal axis hyperactivation, platelet hyperreactivity, and endothelial dysfunction. Selective serotonin reuptake inhibitors and cognitive behavioral therapy demonstrate the strongest evidence for treating depression in cardiac populations. Collaborative, stepped-care, and integrated cardiac rehabilitation models consistently improve psychological outcomes, with variable effects on cardiovascular endpoints. MHDs and ACSs form a self-reinforcing clinical continuum. Routine mental health screening and integrated cardio-psychiatric care represent essential components of secondary prevention and long-term outcome optimization.

## 1. Introduction

Acute coronary syndromes (ACSs) represent a group of closely related ischemic entities that form a continuous clinical spectrum with progressively increasing severity. This spectrum comprises unstable angina, non-ST segment elevation myocardial infarction (NSTEMI), and ST segment elevation myocardial infarction (STEMI) [1]. Across this continuum, patient presentation varies substantially. Some individuals may exhibit minimal or no symptoms, whereas others develop recurrent or persistent chest discomfort accompanied by autonomic or respiratory complaints. Electrocardiographic patterns observed in ACSs are similarly heterogeneous. Tracings may appear normal or demonstrate ischemic changes, including ST segment depression, ST segment elevation, or complex ventricular arrhythmias. Initial evaluation combining clinical features and ECG characteristics allows for provisional classification into non-ST segment elevation ACS (NSTE ACS) or STEMI [2].

At present, ACS constitutes the dominant pathological mechanism underlying cardiovascular mortality on a global scale [3]. Worldwide dissemination of industrialization and urban lifestyles has driven an unprecedented rise in the incidence of acute coronary events, reaching proportions comparable to a global pandemic. Across populations, coronary artery disease (CAD) is responsible for nearly one-third of all deaths, making it the leading individual cause of mortality in adults. Within the United States, a new acute coronary episode is documented roughly every 25 s, while a fatal outcome related to ACS occurs approximately once per minute. CAD and its acute clinical expression as ACS impose substantial consequences at multiple levels. Individuals experience marked reductions in quality of life, healthcare systems face sustained pressure, and societies bear extensive economic costs. Therefore, these disorders represent a central driver of illness and death across diverse ethnicities, cultures, and demographic groups [4].

Beyond the overarching epidemiological impact of ACS, attention has increasingly focused on the complex group of factors that predispose individuals to acute ischemic events. Classical cardiometabolic conditions remain central contributors. These include hyperlipoproteinemia or dyslipidemia characterized by elevated low-density lipoprotein cholesterol, arterial hypertension, diabetes mellitus or metabolic syndrome, and chronic inflammatory states related to infection or autoimmunity. In contrast, higher concentrations of high-density lipoprotein cholesterol are consistently associated with lower coronary risk. Several additional mechanisms meaningfully shape both the likelihood of acute events and subsequent outcomes. Examples include high-risk atherosclerotic plaque morphology, abnormalities of coronary flow with low shear stress, endothelial dysfunction, and coronary microvascular impairment, all of which contribute to plaque instability and ischemic risk. Moreover, persistent or excessive inflammatory activity constitutes a central pathogenic driver. This state is reflected by increased circulating biomarkers such as high-sensitivity C reactive protein, serum amyloid A, interleukin 6, interleukin 1 receptor antagonist, tumor necrosis factor alpha, soluble CD40 ligand, and pregnancy-associated plasma protein A [5,6,7].

In addition to biological mechanisms, converging evidence identifies psychiatric disorders and sustained psychosocial stress as clinically meaningful modifiers of ACS vulnerability. Moreover, individuals with mental health disorders (MHDs) exhibit elevated mortality during the acute phase of ACS and remain at increased risk of death over the subsequent five-year period [8].

Globally, age-standardized, disability-adjusted life-year rates attributable to mental disorders showed little variation from 1990 to 2019. In contrast, the absolute burden rose substantially, with total DALYs increasing by more than fifty percent over this interval [9].

MHDs involve clinically relevant disturbances of cognition, emotion, or behavior that impair functioning. In Western countries, around 10% of adults live with an MHD. Anxiety disorders affect about 359 million people globally, depressive disorders around 280 million, and bipolar disorder approximately 37 million individuals [10,11]. Post-traumatic stress disorder (PTSD) has been experienced by nearly 3.9% of the world population [12]. Schizophrenia affects close to 23 million people and is associated with a life expectancy reduction of roughly nine years [10]. Moreover, individuals with an MHD show reduced survival compared with the general population, with cardiovascular diseases (CVDs) representing a major driver of premature mortality [11].

Patients with heart disease exhibit approximately double the prevalence of anxiety and mood disorders. Increasing levels of psychosocial stress show a graded association with the onset and progression of atherosclerotic cardiovascular disease (ASCVD), independent of traditional risk factors and sex. Depression, anxiety, and chronic psychological distress are consistently linked to poorer cardiovascular prognosis. Identification of depressive symptoms has been associated with fewer major ASCVD events during long-term follow-up. Accordingly, combined psychological and pharmacological strategies may improve outcomes and should be considered in ACS patients presenting with depression, anxiety, or stress [2,13].

Interactions between MHDs and CVDs operate in a reciprocal and multifaceted manner. Patients with established cardiovascular disease display heightened vulnerability to developing psychiatric conditions as a consequence of chronic physical and emotional stress. Conversely, individuals with pre-existing mental disorders, especially depression and anxiety, demonstrate a higher likelihood of developing ACS. Together, these observations underscore the importance of an integrated perspective, since adverse events in psychological and cardiovascular health frequently potentiate each other, contributing to adverse clinical outcomes and substantial healthcare burden [14].

Despite robust evidence supporting the link between MHDs and CVDs, the biological and behavioral pathways underlying this association remain incompletely elucidated. Cardiovascular risk prediction models (e.g., GRACE, TIMI, and SCORE2) do not incorporate psychiatric variables despite consistent epidemiologic evidence demonstrating independent prognostic impact, representing a translational gap between observational findings and clinical implementation [15,16,17].

Although the existing literature has examined depression and psychosocial stress in coronary heart disease, most investigations have focused on single diagnostic categories, have evaluated either incident cardiovascular risk or post-event prognosis in isolation, and have rarely integrated mechanistic, prognostic, and health system disparities within a unified bidirectional framework [18,19,20,21]. Moreover, psychiatric conditions have been predominantly examined isolated entities. Consequently, there remains a lack of integration regarding the full range of relevant disorders, including PTSD, bipolar disorder, schizophrenia, and suicidality into a synthesized bidirectional approach.

The present narrative review aims to critically integrate and contextualize current evidence on the bidirectional relationship between MHDs and ACSs, with emphasis on shared biological mechanisms, prognostic implications, and therapeutic integration. Furthermore, it stratifies the evidence by targeting a broader spectrum of MHDs and explicitly characterizes the bidirectionality of the MHD–ACS relationship by differentiating incident ACS risk from post-ACS outcomes, addressing reverse causation, integrating mechanistic pathways at the molecular, autonomic, endothelial, and platelet levels, and addressing therapeutic translation within a single conceptual continuum.

## 2. Methodology of Research

The present narrative review was designed to integrate peer-reviewed scientific evidence and authoritative institutional resources in order to provide a comprehensive and clinically oriented synthesis of the bidirectional relation between MHDs and ACSs, encompassing epidemiology, pathophysiological mechanisms, clinical outcomes, and management implications. A narrative design was adopted to integrate heterogeneous evidence and conceptual perspectives relevant to the mental health–cardiovascular interface. Therefore, no quantitative synthesis was performed, and the review provides a structured qualitative evaluation of the literature.

An extensive search of major electronic databases was systematically performed, including PubMed, Web of Science Core Collection, ScienceDirect, and SpringerLink. In addition, official documents and web-based resources from international health authorities and professional societies (e.g., World Health Organization, American Heart Association, American College of Cardiology, and European Society of Cardiology) were consulted to ensure inclusion of current clinical guidelines and policy-relevant evidence.

**Figure 1:**
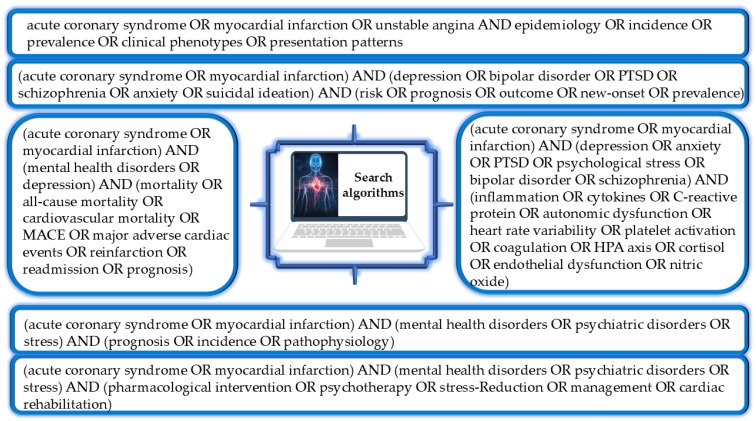
Representative Boolean search algorithms for identifying the literature on mental health disorders and acute coronary syndromes, including bidirectional associations. PTSD, post-traumatic stress disorder; MACE, major adverse cardiac event; HPA, hypothalamic–pituitary–adrenal (axis).

Predefined search algorithms were constructed for each thematic domain using combinations of controlled vocabulary (MeSH/Emtree) and free-text keywords linked by logical operators (Figure 1). The most representative keywords included: “acute coronary syndrome”, “myocardial infarction”, “unstable angina”, “depression”, “anxiety”, “post-traumatic stress disorder”, “bipolar disorder”, “psychological stress”, “mental health”, “mortality”, “cardiovascular mortality”, “major adverse cardiac events”, “reinfarction”, “readmission”, “prognosis”, “inflammation”, “cytokines”, “heart rate variability”, “platelet activation”, “coagulation”, “HPA axis”, “cortisol”, “endothelial dysfunction”, “nitric oxide”, “medication adherence”, “cardiac rehabilitation”, and “screening”. Search strategies incorporated logical operators and truncation to maximize retrieval accuracy. Bibliographic lists of selected articles were manually reviewed, and relevant publications were subsequently selected for inclusion in the final reference set.

**Figure 2:**
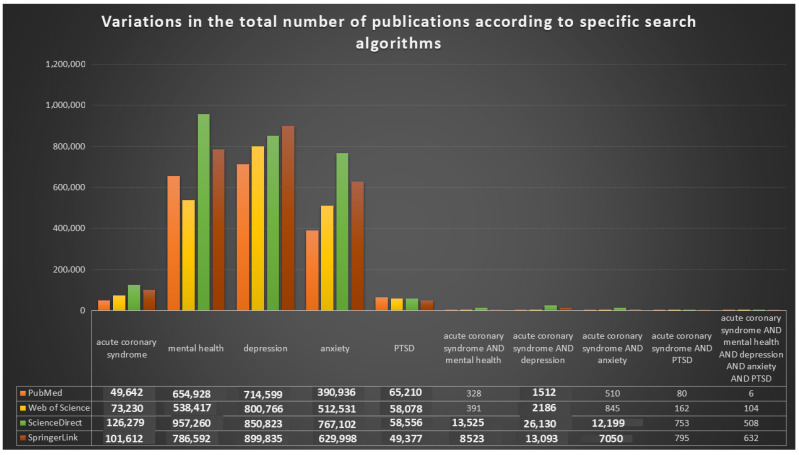
Distribution of publications related to acute coronary syndrome, mental health disorders, and their combined association identified in major biomedical databases using Boolean search strategies.

Following this structured approach, an exploratory bibliometric screening was performed to estimate research volume using broad logical operators combining the terms “acute coronary syndrome”, “mental health”, “depression”, “anxiety”, and “post-traumatic stress disorder” (Figure 2). This preliminary mapping revealed that while extensive bodies of the literature exist for ACS and mental health as separate fields, comparatively and significantly few publications explicitly examine their combined or interacting effects, highlighting a persistent knowledge gap and supporting the need for integrated, bidirectional investigation of the mental health–ACS relationship.

Eligible sources were restricted to English-language publications and included original research articles, narrative and systematic reviews, meta-analyses, consensus statements, clinical guidelines, correspondences, and authoritative reports addressing mental health disorders, psychosocial stress, and their associations with ACS or atherosclerotic cardiovascular disease. Studies focusing on epidemiology, biological, and behavioral mechanisms, prognostic impact, and therapeutic or preventive interventions were prioritized. Exclusion criteria comprised non-English publications, duplicate records, conference abstracts without full text, and any type of documents lacking sufficient scientific rigor or relevance to the MHD–ACS relationship.

No strict publication timeframe was imposed. Although this narrative review does not follow a formal systematic review protocol, emphasis was placed on recent high-quality evidence, including systematic reviews, meta-analyses, and large prospective cohort studies. Final source selection was guided by relevance, methodological quality, and contribution to elucidating the bidirectional relationship between mental health and ACS. Overall, 250 references were included to support the data presented in this review.

## 3. Epidemiology and Clinical Phenotypes in MHD–ACS

The epidemiological relationship between MHDs and ACSs can be analytically differentiated into three clinically distinct domains: (1) mental health disorders as predictors of incident ACS, (2) mental health disorders as determinants of post-ACS prognosis, and (3) ACS as a precipitant of subsequent psychiatric morbidity. Although these domains frequently overlap, maintaining this temporal distinction is essential to prevent conflation between etiologic susceptibility, post-event prognostic modification, and secondary psychiatric sequelae. Mechanistic pathways are synthesized in Section 4, while the present subsection focuses on epidemiology and prognostic association.

### 3.1. Prevalence Across the ACS Continuum

Psychiatric phenotypes are frequently observed throughout the ACS trajectory, from acute hospitalization to long-term follow-up, with prevalence varying by disorder category, timing of evaluation, and individual patient features [22]. Available evidence indicates that individuals with CVD frequently exhibit clinically relevant anxiety and depressive symptoms, with reported prevalence rates generally ranging between approximately twenty and forty-five percent [3].

A large retrospective cohort of approximately three million individuals, including about one percent with serious mental illness (SMI), evaluated patterns of care and outcomes relative to matched controls. Within the ACS population, patients with SMI were younger, more frequently female, and showed higher prevalence of diabetes and obesity than those without SMI [23].

Using a nationwide Swedish registry, all individuals with schizophrenia who developed acute myocardial infarction between 2000 and 2018 were identified and compared with a large cohort of acute myocardial infarction patients without schizophrenia. Patients with schizophrenia experienced acute myocardial infarction roughly a decade earlier, with a median age in the mid-sixties versus early seventies in controls, and showed higher rates of diabetes, heart failure, and chronic obstructive pulmonary disease. Consequently, this population exhibited more than a twofold increase in major adverse cardiovascular events and all-cause mortality [24].

A nationwide Danish cohort including over twelve thousand STEMI patients undergoing primary PCI identified approximately four percent who fulfilled criteria for SMI. Within this population, SMI was associated with a more unfavorable cardiovascular risk profile and poorer clinical outcomes. Patients with SMI exhibited higher rates of active smoking, greater prevalence of diabetes, and modestly prolonged symptom to reperfusion intervals compared with those without SMI. Although in hospital treatment strategies were largely comparable, SMI remained independently linked to progressively increased rates of major adverse cardiac events during follow-up. This excess risk, which rose over time, was driven predominantly by higher mortality [25].

A meta-analytic synthesis of six mortality studies including nearly six hundred thousand ACS patients showed that SMI is associated with a modest but significant increase in post ACS mortality. Even when restricted to cohorts composed exclusively of ACS cases, an excess risk of approximately ten percent persisted. At the population level, individuals with psychiatric disorders exhibit markedly higher cardiovascular death rates, with increased age-standardized mortality and almost double standardized mortality for ischemic heart disease and AMI. These findings indicate that mental illness confers elevated cardiovascular risk prior to ACS presentation. In parallel, inequalities extend to post ACS care. Pooled evidence from multiple analyses demonstrates that patients with mental illness undergo cardiac procedures at substantially lower rates than those without psychiatric conditions [26].

Another meta-analytic evaluation confirmed that SMI is prevalent among ACS populations and is consistently associated with excess mortality and disparities in treatment delivery throughout the post-ACS course. Pooled data from more than twenty studies encompassing over twelve million ACS patients indicated an overall SMI prevalence of approximately four percent. Across analyses, SMI was linked to substantially higher mortality after ACS, with elevated risk evident early and increasing over time. Thirty-day mortality was notably higher, while one-year mortality showed an even greater excess. First ACS presentation appeared to confer particularly pronounced vulnerability. Schizophrenia demonstrated persistently increased mortality, whereas BD showed no overall excess risk but markedly higher mortality at incident ACS, indicating phenotype specific patterns. Along the continuum of care, SMI was associated with significantly reduced use of invasive strategies, including coronary angiography, percutaneous coronary intervention, and coronary artery bypass grafting. In addition, patients with SMI were less likely to receive cardioprotective pharmacotherapy within the first year, particularly angiotensin-converting enzyme inhibitors or angiotensin receptor blockers, beta-blockers, and statins [27].

Shao et al. evaluated the prognostic significance of schizophrenia across both the acute phase and extended course of ACS, identifying persistently inferior survival relative to ACS patients without psychiatric illness. A pooled synthesis of nine longitudinal cohorts, encompassing more than three and a half million hospitalized ACS cases, revealed a markedly elevated, multivariable adjusted risk of death among individuals with schizophrenia. Schizophrenia was associated with an increase in post-ACS mortality approaching seventy percent, confirming a substantial and durable adverse prognostic effect [28].

Population level data indicate that psychological distress is common after ACS and constitutes a substantial yet frequently overlooked component of post-event morbidity. Nearly one in five ACS patients meet criteria for clinically relevant psychological distress. Pooled symptom-specific estimates were even higher, with anxiety and depressive symptoms affecting roughly one-third of patients, PTSD present in more than one-tenth, and fear of death reported by almost half. The highest burden occurs during hospitalization, when anxiety, depression, and PTSD reach their greatest prevalence, highlighting an early period of heightened vulnerability [29].

#### 3.1.1. MHDs as Predictors of Incident ACS

Psychological determinants are increasingly implicated in the pathogenesis of coronary heart disease. In individuals experiencing a first ACS event, psychiatric morbidity was substantially more frequent than in non-ACS populations, with odds exceeding fourfold for the presence of any mental disorder [30]. These data support MHDs as upstream modifiers of first-ACS susceptibility rather than solely post-event sequelae.

#### 3.1.2. MHDs as Determinants of Post-ACS Prognosis

The disorder-specific sections that follow (Section 3.2, Section 3.3, Section 3.4, Section 3.5, Section 3.6 and Section 3.7) primarily address the impact of MHDs on post-ACS prognosis, including mortality, recurrent ischemic events, treatment disparities, and adherence-related mechanisms, given their direct relevance to secondary prevention and long-term outcomes. Accordingly, etiologic evidence for incident ACS risk is conceptually separated from prognostic evidence after ACS to avoid conflating susceptibility with post-event clinical trajectories.

#### 3.1.3. ACS as a Precipitant of Subsequent Psychiatric Morbidity

Longitudinal observations reveal a substantial and enduring psychiatric burden following ACS. In a prospective cohort of ACS patients, nearly half met criteria for major depressive disorder at six months, with significant persistence from hospital discharge through three- and six-month assessments. Symptom intensity peaked around three months and showed only partial subsequent improvement. Anxiety-related conditions were likewise frequent at six months, encompassing social phobia, PTSD, panic disorder, specific phobia, obsessive–compulsive disorder, agoraphobia, and dysthymia [31]. This post-event psychiatric burden is clinically relevant because it can persist beyond discharge and may influence adherence, rehabilitation engagement, and long-term outcomes.

### 3.2. Depression–ACS

Among psychiatric conditions observed after ACS, depressive disorders emerge as one of the most prevalent phenotypes and are consistently linked to worse cardiovascular outcomes during follow-up. In a cohort of 315 patients, more than half of hospitalized ACS patients met criteria for depressive disorder and experienced nearly double the rate of cardiovascular complications at twelve months compared with those without depression [32].

Across consistent studies of ACS and CVD populations, reported prevalence estimates for depression have ranged approximately between 15.6% and 31.3% [33,34]. Moreover, meta-analytic evidence indicates that roughly one-fifth of individuals with ACS develop major depressive disorder. In addition, nearly two-thirds continue to exhibit clinically relevant depressive symptoms months after the acute event. In the same analysis, pooled estimates showed depression prevalence of approximately fourteen percent for mild severity, twelve percent for moderate severity, and fifteen percent for severe forms [3].

Carney et al. reported that both incident and recurrent major depressive episodes are linked to significantly poorer survival following MI when compared with patients without depression. Individuals developing depression for the first time after MI exhibited worse outcomes than those with a prior depressive history, suggesting a stronger adverse prognostic impact of new onset depression. Together, these observations indicate that the temporal pattern and clinical background of depression define distinct post-ACS phenotypes with differing prognostic relevance, with first-episode depression representing a particularly high-risk subgroup requiring heightened clinical attention and follow-up [35].

Depression occurring after myocardial infarction has consistently been linked to adverse prognosis. Compared with nondepressed individuals, affected patients show approximately 1.6- to 2.7-fold higher risk of early mortality or recurrent cardiovascular events [36,37]. Nevertheless, depression retains an independent prognostic effect, conferring an approximately twenty-two percent higher risk of all-cause mortality and a thirteen percent higher risk of cardiovascular events for each standard deviation increase in depression severity [36]. Evidence indicates that post-ACS depression is not a uniform entity but comprises clinically distinct phenotypes determined by onset and trajectory. Both recurrent and first-episode major depression are associated with reduced survival after acute MI, although new onset episodes demonstrate a stronger prognostic impact than recurrent depression [35,38,39].

Evidence from two large population-based cohorts in China shows that adults with depression have markedly higher mortality than individuals without depressive symptoms, including a clearly increased risk of cardiovascular death and with particularly pronounced excess risk observed among men. Collectively, these findings indicate that depression extends beyond a psychiatric diagnosis and represents a clinically relevant contributor to premature cardiovascular mortality, supporting its role as a meaningful risk factor for the development and progression of CVD [40].

Depression in patients after myocardial infarction has been linked to poorer adherence to recommended lifestyle modification and reduced compliance with prescribed therapies. Despite these consequences, depression remains frequently underdiagnosed and undertreated and is often regarded as a secondary consequence of CVD rather than a modifiable cardiovascular risk factor [41].

Beyond biological mechanisms, several behavioral and health system factors contribute significantly to the worsened prognosis observed in patients with coexisting depression and coronary artery disease. Depression is often accompanied by apathy, anhedonia, and a diminished sense of urgency, all of which might delay the recognition and reporting of cardiac symptoms. Depressed patients are more likely to present late after the onset of ACS symptoms, reducing the window for optimal reperfusion therapies. Patients with depression demonstrate markedly lower adherence to secondary prevention strategies, including antiplatelet therapy, statins, beta-blockers, ACE inhibitors, and possibly heart failure treatment [14,42].

Although screening for depression has been recommended in patients with coronary heart disease, early evaluations of proposed protocols revealed suboptimal implementation. Nevertheless, detection of depressive symptoms after acute myocardial infarction represents an important opportunity to enhance comprehensive care and address a major comorbidity. Given the bidirectional relationship between depression and CVD, reciprocal screening of both cardiac and psychiatric populations is essential [43].

### 3.3. Anxiety–ACS

Prior investigations have established depression as an independent predictor of adverse outcomes in coronary heart disease. In contrast, the prognostic significance of anxiety, especially among patients with ACS, remains insufficiently defined [19,44]. Anxiety has been proposed as a prognostic risk factor in ACS [45,46], although results across studies are partly inconsistent [44].

Post-myocardial infarction anxiety has been linked to increased risk of long-term cardiac complications and mortality. In up to twenty-five percent, symptom severity is comparable to that observed in psychiatric inpatients. Anxiety frequently persists beyond the acute event. Many patients who report anxiety shortly after ACS continue to display clinically significant symptoms for as long as two years. Generalized anxiety disorder has been identified in nearly one-quarter of individuals with cardiac disease. Panic disorder is also markedly over-represented in cardiovascular populations, with active symptoms reported in a substantial proportion of patients [47].

Anxiety frequently accompanies the acute presentation of ACS and, when considered as a distinct phenotype, carries meaningful prognostic significance. In a large Korean cohort followed over the long term, nearly twenty-five percent of hospitalized ACS patients met the criteria for case-level anxiety. Anxiety during the acute phase independently predicted increased risk of subsequent adverse cardiovascular outcomes, including cardiac mortality, recurrent MI, and repeat revascularization. Furthermore, this relationship was evident primarily among patients with greater initial clinical severity, defined by a Killip class of at least one, in whom anxiety was associated with an almost twofold higher risk of composite cardiovascular events over extended follow-up [48].

In a recent Egyptian ACS cohort, more than one-third of hospitalized patients screened positive for clinically relevant anxiety, and roughly twenty percent reported moderate to severe symptoms. Anxiety showed independent associations with elevated heart rate, hypertension, prior depressive history, and multiple concomitant medications, reflecting a close relationship with cardiovascular risk burden and disease complexity. Importantly, anxiety in cardiac populations has been repeatedly linked to unfavorable cardiovascular outcomes, including higher rates of recurrent MI and cardiovascular mortality [49].

Li et al. reported that ACS patients presenting with baseline anxiety exhibit a modest but significant increase in mortality risk and a substantially higher likelihood of major adverse cardiac events (MACEs) compared with nonanxious individuals. Subgroup analyses indicated that coexisting depression meaningfully modifies this association, suggesting important interaction effects. Given the strong overlap between anxiety and depression in ACS populations, depressive symptoms may represent a more powerful determinant of prognosis than anxiety alone [44].

Nevertheless, a bidirectional relationship is also evident in this context. Anxiety has been identified as an independent predictor of incident coronary artery disease and cardiac mortality among initially healthy populations. Prospective data indicate an approximately twenty-six percent increase in risk of developing coronary artery disease associated with anxiety, along with a forty-eight percent higher risk of cardiac death. Although the magnitude of this association appears somewhat weaker than that observed for depression, it remains clearly stronger than the effect attributed to anger [50].

### 3.4. PTSD–ACS

This PTSD is identified when exposure to a life-threatening traumatic experience is followed by a characteristic group of symptoms. These include intrusive recollections, avoidance of trauma-related cues, persistent negative changes in mood and cognition, and heightened arousal or reactivity [51]. PTSD may emerge following cardiac events and is also linked to elevated cardiovascular risk. In addition, PTSD arising from non-medical trauma has been associated with higher incidence of CHD among previously healthy individuals [41,51].

Findings from the past decade support a robust and independent relationship between PTSD and first-onset CVD and suggest a probable association with recurrent cardiovascular events. PTSD arising from traumatic life experiences confers higher risk for developing CVD, while exposure to life-threatening cardiovascular events may precipitate PTSD and further amplify the likelihood of recurrent CVD, underscoring a bidirectional link [52].

A meta-analytic investigation demonstrated that PTSD represents a frequent and clinically meaningful sequela of ACS with important prognostic implications. Across longitudinal cohorts, the presence of PTSD symptoms was associated with nearly a twofold increase in the risk of subsequent cardiac events and or death compared with patients without PTSD. Moreover, these relationships persisted after adjustment for conventional cardiovascular risk factors and indices of disease severity. These observations support PTSD as a distinct post-ACS phenotype linked to unfavorable prognosis and highlight the importance of routine detection and tailored intervention within ACS care pathways [53].

Multiple determinants of ACS-related PTSD have been identified, spanning contextual, individual, and relational domains during the emergency care period. Within the emergency department setting, overcrowding, prolonged delays before evaluation, and exposure to invasive or life sustaining procedures are associated with heightened psychological distress and greater subsequent PTSD symptom burden. Patient perceived threat during the acute event, particularly intense fear of death, loss of control, and marked anxiety, shows a stronger association with later PTSD than objective indices of cardiac injury [51].

Prospective observations in another emergency care setting indicate that close to one-third of individuals presenting with ACS develop PTSD within the first month after the event. Elevated perceived stress at initial presentation independently predicted subsequent PTSD onset. Patients with ACS who manifested PTSD reported chest pain recurrences more often and demonstrated poorer adherence to prescribed cardiovascular therapies, linking post-traumatic symptomatology to unfavorable health behaviors and outcomes [54].

Evidence derived from large cardiac rehabilitation cohorts indicates that ACS can trigger formally diagnosed PTSD in roughly one-tenth of patients, with prevalence approaching sixteen percent when symptom-based screening instruments are applied. Individuals who develop PTSD after ACS exhibit diminished exercise performance, reaching a smaller proportion of their predicted maximal workload, consistent with lower habitual physical activity and potential downstream cardiovascular risk. Prior investigations further associate post-ACS PTSD with increased rates of recurrent cardiac events and mortality [55].

Within the REACH cohort, 830 individuals assessed for possible ACS completed event-focused PTSD evaluations one month after their emergency presentation, with symptoms anchored to the perceived cardiac threat and hospitalization experience. Despite definitive ACS being confirmed in only about one-third of cases, PTSD symptom patterns and scoring properties were comparable between confirmed and excluded ACS, across sexes, and between English- and Spanish-speaking participants [56].

### 3.5. Schizophrenia–ACS

Schizophrenia represents a severe mental illness that confers additional prognostic burden when it coexists with acute coronary syndromes. Epidemiological data indicate that people with schizophrenia exhibit markedly higher rates of cardiovascular disease than the general population. This vulnerability is largely explained by clustering of adverse cardiometabolic traits, including obesity, dysglycemia, metabolic syndrome, and tobacco use. Among patients experiencing acute myocardial infarction, the presence of schizophrenia has been associated with significantly increased all-cause mortality compared with individuals without this diagnosis. Moreover, several studies report lower utilization of invasive coronary therapies in this population. Reduced access to procedures such as percutaneous coronary intervention and coronary artery bypass grafting may contribute to the observed outcome disparities, despite these strategies being central components of contemporary myocardial revascularization and secondary prevention [41,57,58].

Evidence from a systematic review of fourteen investigations indicates that individuals with schizophrenia display persistently poorer clinical trajectories following ACS. Adverse outcomes include excess early and late mortality, higher rates of major cardiovascular complications, cerebrovascular events, and hemorrhagic episodes when compared with patients without schizophrenia. Survival gains observed in the general ACS population over recent decades have not been mirrored in this subgroup. Elevated in-hospital and 30-day mortality further underscore the magnitude of risk. These unfavorable patterns appear to arise from multiple interacting mechanisms. Contributing factors include accumulation of traditional cardiovascular risk determinants, frequent metabolic comorbidity, lifestyle behaviors, and pronounced disparities in ACS care delivery [59].

An analysis of individuals hospitalized with ACS demonstrated that comorbid schizophrenia confers a substantially amplified risk of adverse cardiovascular outcomes. Compared with carefully matched controls without psychiatric illness, patients with schizophrenia experienced a sixty percent higher incidence of major adverse cardiac events, more than a twofold increase in overall mortality, and a significantly elevated likelihood of stroke. By contrast, rates of recurrent infarction and duration of hospitalization were broadly comparable between groups. Individuals with schizophrenia carried a disproportionate load of noncardiac medical comorbidities, including diabetes mellitus, heart failure, cardiomyopathy, chronic obstructive pulmonary disease, and anemia. Conversely, lower documented prevalence of hypertension and dyslipidemia was observed, a pattern consistent with under-recognition and suboptimal management of conventional cardiovascular risk factors [60].

Pooled evidence derived from very large ACS populations indicates that a pre-existing diagnosis of schizophrenia confers a substantial survival disadvantage following an acute coronary event. Across datasets encompassing more than three and a half million individuals, patients with schizophrenia exhibited an almost seventy percent higher likelihood of death after ACS compared with those without psychiatric illness. Notably, the mortality excess is evident during the early post-event period and remains pronounced over extended follow-up. This unfavorable association persists even among patients who undergo coronary revascularization [28].

A very large meta-analytic dataset encompassing more than three million patients hospitalized for acute myocardial infarction indicates profound disparities in the delivery of invasive coronary care among individuals with schizophrenia. Even after extensive adjustment for patient characteristics, comorbidities, and institutional factors, this population was substantially less likely to undergo coronary revascularization compared with patients without psychiatric disorders. Overall, utilization of revascularization procedures was reduced by nearly fifty percent, with pronounced underuse of both percutaneous coronary intervention and coronary artery bypass surgery. Such systematic treatment gaps offer a credible clinical mechanism for the persistently elevated mortality observed after ACS in schizophrenia and underscore structural inequities in access to evidence-based acute cardiac therapies for this high-risk group [61].

### 3.6. BD-ACS

BD characterized by alternating manic and depressive episodes constitute a group of persistent psychiatric disorders with substantial clinical impact [62]. Population-level data demonstrated that individuals diagnosed with BD who present with acute coronary syndrome experience substantially poorer clinical outcomes than comparable patients without psychiatric illness. This group exhibited a roughly forty percent increase in major adverse cardiovascular events, more than a seventy percent elevation in overall mortality, and nearly double the risk of cerebrovascular events, whereas rates of recurrent myocardial infarction were slightly lower. At baseline, patients with BD more frequently carried comorbid heart failure, valvular pathology, anemia, chronic obstructive pulmonary disease, and a history of stroke. Together, these findings indicate a pronounced cardiovascular risk burden and identify BD as a clinically relevant determinant of prognosis following ACS [63].

Among 171 individuals admitted for ACS, BD was identified in 11.7% and represented nearly half of all detected mood disorders. Unlike major depressive disorder, BD independently correlated with younger age at first coronary presentation and with a greater burden of prior ischemic events. Patients with BD frequently exhibited activation and mixed-state characteristics, including psychomotor excitation and mood lability, suggesting that bipolarity is prevalent in ACS and defines a cardiovascular risk profile distinct from unipolar depression [64].

Across Danish nationwide registries spanning 1996 to 2016, 497 individuals with BD hospitalized for ACS were matched in a 1:2 ratio with psychiatric healthy comparators. Patients with BD consistently experienced deficiencies in cardiac care. Moreover, patients with BD were less likely to obtain recommended secondary prevention pharmacotherapy after ACS. Only antiplatelet therapy with acetylsalicylic acid, lipid-lowering agents, and beta-blockers exhibited a modest reduction in disparity. Importantly, these limited gains did not translate into narrowing of the one-year all-cause or presumed cardiovascular mortality differences [65].

Among adults diagnosed with BD, Etxaniz-Oses et al. documented a substantially more adverse cardiometabolic phenotype compared with psychiatrically healthy individuals. Combined abnormalities translated into higher predicted cardiovascular risk according to SCORE2 and a vascular age exceeding chronological age. Collectively, metabolic, inflammatory, and fitness-related derangements associated with BD are likely to heighten susceptibility to CAD and, consequently, increase the probability of future ACS [66].

In individuals experiencing a first ACS event, affective and stress-related symptoms are common and display substantial clinical variability. The considerable convergence between type A traits, demoralization, and depressive symptomatology, patterns that resemble subthreshold bipolar-spectrum characteristics, supports the hypothesis that affective instability related to bipolar vulnerability and somatically weighted depressive presentations may constitute relevant mechanistic links between BD liability and unfavorable prognosis following ACS [67].

### 3.7. Suicidal Ideation–ACS

Suicidality represents one of the most severe and insufficiently addressed psychiatric sequelae in individuals surviving ACS. Meta-analytic data encompassing more than 430,000 participants indicate that exposure to ACS confers a substantially elevated likelihood of suicidal ideation relative to non-ACS populations, with an estimated 45% relative increase in risk. Approximately one in seven survivors report suicidal thoughts, and about 1% engage in suicide attempts, with vulnerability peaking during the first six months after the index event [68].

Complementary real-world evidence further confirms this temporal clustering of suicide risk after ACS. Population-based investigations demonstrate that patients diagnosed with ACS experience significantly higher rates of suicide compared with matched controls, with the greatest excess risk occurring within the first half-year after diagnosis [69]. A large case-referent analysis additionally shows that the association between ACS and suicide persists even after controlling for depression, pre-existing psychiatric illness, and major medical comorbidities and remains detectable for several years, underscoring ACS as an independent and durable vulnerability state for suicidality [70].

Longitudinal cohort data provide further mechanistic insight. Suicidal ideation is common in the immediate post-ACS period and, although it declines over time, remains present in a meaningful proportion of patients at one year. Importantly, early post-event suicidality appears partly biologically modulated. Carriers of the short allele of the serotonin transporter promoter polymorphism (5-HTTLPR) exhibit increased odds of suicidal ideation during the acute phase independent of depression severity or cardiac status, whereas this genetic effect is no longer evident at later follow-up [71].

### 3.8. Methodological Considerations and Sources of Heterogeneity

The interpretation of the MHD–ACS association requires careful attention to diagnostic heterogeneity, measurement variability, illness severity, treatment exposure, and residual confounding. To minimize diagnostic conflation, Section 3.2, Section 3.3, Section 3.4, Section 3.5, Section 3.6 and Section 3.7 were intentionally structured as disorder-specific subsections, allowing distinct epidemiologic and prognostic patterns to be evaluated separately.

First, MHDs are biologically and clinically heterogeneous entities. Major depressive disorder, anxiety disorders, PTSD, schizophrenia, and bipolar disorder differ in neurobiological substrates, inflammatory profiles, cardiometabolic burden, and pharmacologic exposure. Pooling these conditions without stratification risks obscuring phenotype-specific cardiovascular patterns [50,72,73,74].

Second, substantial variability exists in exposure assessment across studies. Many investigations rely on symptom-based screening instruments such as the Patient Health Questionnaire-9 (PHQ-9), a validated tool for depressive symptom severity but not equivalent to structured diagnostic interviews [75,76]. Clinical diagnoses derived from Diagnostic and Statistical Manual of Mental Disorders-based interviews represent a higher diagnostic threshold, whereas administrative datasets based on International Classification of Diseases coding may underestimate milder or untreated cases and are susceptible to misclassification bias [77,78]. These methodological differences influence prevalence estimates and effect sizes and complicate cross-study comparisons.

Third, severity, chronicity, and temporal onset appear to modify cardiovascular risk. Incident post-ACS depression has been associated with worse survival compared with recurrent depression, suggesting prognostic heterogeneity based on temporal pattern [79]. Moreover, graded increases in depressive symptom severity correlate with stepwise elevations in cardiovascular events and mortality [80]. Treatment status further modifies risk, as antidepressant exposure and symptom remission may partially attenuate adverse outcomes [81].

Fourth, observational studies are inherently vulnerable to confounding. Smoking prevalence is markedly elevated among individuals with severe mental illness [82,83], and clustering of metabolic syndrome components is well documented in psychiatric populations [84]. Socioeconomic disadvantage independently contributes to cardiovascular risk and may mediate part of the observed association by impacting also MHDs [85,86]. Additionally, depression is consistently associated with reduced medication adherence [87], potentially influencing post-ACS prognosis independently of biological mechanisms.

Beyond confounding, reverse causality is a recurrent threat in observational MHD–ACS research and requires explicit temporal handling. Subclinical or emerging cardiovascular disease may contribute to depressive symptoms, inflating associations. In the prospective Nurses’ Health Study, women with baseline CVD/stroke were excluded, depression was assessed repeatedly, and sensitivity analyses excluding participants who developed intervening nonfatal CVD events did not materially change results, arguing against symptomatic CVD during follow-up as the primary explanation for the observed association [88].

Similar efforts to address reverse causality are evident in other large prospective cohorts. A related methodological challenge in longitudinal psychiatric–cardiovascular research is the possibility that early subclinical atherosclerosis may precede and influence depressive symptomatology. In the CARDIA cohort, this concern was partially addressed through clear temporal sequencing: participants were free of clinical cardiovascular disease and had no coronary artery calcification (CAC) at baseline, depressive symptoms were measured at Year 15, and incident CAC was defined as new calcification detected five years later. Higher total depressive symptoms predicted CAC onset, with partial attenuation after adjustment for tobacco use and mean arterial pressure. Nevertheless, because several cardiovascular risk factors were measured concurrently with depressive symptoms, residual reverse causality and confounding cannot be fully excluded [89].

In the Mass General Brigham Biobank retrospective cohort, Civieri et al. reduced this risk by restricting exposure to anxiety/depression diagnosed before the 10-year baseline, excluding new post-baseline diagnoses, and excluding participants with pre-baseline MACEs. Within this temporally anchored design, pre-existing anxiety/depression predicted incident cardiometabolic risk factors, and these risk factors partly mediated the association with subsequent MACEs. However, reliance on International Classification of Diseases-based ascertainment and potential detection/ascertainment bias mean that residual reverse causality and confounding remain possible [90].

To move beyond these inherent constraints of observational designs, genetically informed approaches have been increasingly applied to clarify directionality at the level of inherited liability. Genetically informed designs provide an additional framework for strengthening causal inference beyond conventional observational cohorts. Mendelian randomization (MR) leverages germline genetic variants associated with an exposure as instrumental variables, exploiting their random allocation at conception to reduce confounding and minimize reverse causality. By anchoring exposure to inherited genetic liability rather than measured symptom status, MR offers a temporally robust approach to evaluating directionality within the MHD–ACS relationship [91].

Lu et al. conducted a two-sample MR and mediation analysis using Genome-Wide Association Study (GWAS) summary statistics from 807,553 individuals for depression and 184,305 individuals (60,801 CAD cases) for coronary outcomes. Genetic liability to depression was causally associated with a 14% higher risk of CAD (OR 1.14, 95% CI 1.06–1.24) and a 21% higher risk of MI (OR 1.21, 95% CI 1.11–1.33), with type 2 diabetes and tobacco smoking as significant mediators. The reverse MR, treating genetic liability to CAD as exposure and depression as outcome, yielded no significant causal effect across all sensitivity analyses [92].

Li et al. independently evaluated bidirectional causality between depression and cardiovascular diseases in a two-sample MR study. Genetically doubling the odds of depression was causally associated with increased risk of CAD (OR 1.099, 95% CI 1.031–1.170) and MI (OR 1.146, 95% CI 1.070–1.228), with blood lipid levels and smoking as mediators. Critically, no causal association was observed in the reverse direction, from CVD to depression [93].

Xu et al. confirmed a unidirectional causal relationship between major depressive disorder and CHD in an independent bidirectional MR study using IEU Open GWAS data. The forward analysis (MDD → CHD) reached significance using inverse-variance weighted, MR-Egger, and weighted median methods, while the reverse analysis (CHD → major depressive disorder) did not, leading the authors to conclude that there is insufficient evidence that CHD causally increases the risk of major depressive disorder at the genetic liability level [94].

Across all four independent MR analyses, employing different GWAS datasets, instrumental variable sets, and sensitivity methods, the directional signal is consistent: genetic liability to depression confers causal risk for coronary disease, while genetic liability to coronary disease does not causally predict depression.

Collectively, these genetically informed analyses converge toward a predominantly forward causal direction from depression liability to coronary disease. Although MR cannot fully exclude horizontal pleiotropy or phenotype misclassification, it strengthens temporal inference and reduces the likelihood that the observed association is solely attributable to reverse causality. At present, genetically informed evidence remains substantially more developed for depression than for other psychiatric phenotypes, and comparable MR data for anxiety disorders, PTSD, bipolar disorder, or schizophrenia in relation to ACS are still limited. This imbalance should be considered when extrapolating causal interpretations across the broader spectrum of mental health disorders.

Although many large cohorts adjust for traditional cardiovascular risk factors [19,95], residual confounding cannot be fully excluded in observational research [96,97]. Consequently, while the consistency of associations across diverse populations strengthens biological plausibility, causal inference must remain cautious.

Table 1 summarizes the prevalence, directionality, and prognostic impact of major mental health phenotypes across the ACS continuum, integrating evidence from large observational cohorts and meta-analyses.

Collectively, the evidence supports a bidirectional MHD–ACS relationship across the full clinical continuum. Mental health disorders contribute to incident coronary risk and may worsen post-ACS prognosis through behavioral, biological, and health system mechanisms. Conversely, ACS frequently precipitates subsequent depressive, anxiety, trauma-related symptoms, and suicidality, defining clinically relevant post-event phenotypes. These converging data support systematic mental health screening in ACS and reinforce the need for integrated cardio-psychiatric models to improve secondary prevention and long-term outcomes.

In parallel, the COVID-19 era represented a context in which infection-related biology and pandemic-mediated stressors jointly reshaped ACS presentation, care pathways, and functional recovery.

### 3.9. COVID-19-Linked ACS Dynamics

A growing body of the literature suggests that the COVID-19 era was accompanied by clinically meaningful shifts in ACS risk and care patterns, driven by both infection-related and pandemic-mediated mechanisms. In infected patients, systemic inflammation and a prothrombotic milieu, characterized by elevated cytokines, CRP, troponin, and D-dimer, have been repeatedly observed and may plausibly promote endothelial dysfunction and oxygen supply–demand mismatch, thereby precipitating ACS. Concurrently, lockdowns and health system strain were associated with substantial reductions in ACS admissions (particularly NSTEMI/unstable angina) and decreased use of invasive procedures, raising concerns about delayed presentation and underdiagnosis [98].

A cross-sectional study examining psychological responses to COVID-19 found that acute stress was highly prevalent during active infection, with 40% of patients demonstrating elevated stress levels on the National Stressful Events Survey Acute Stress Disorder Short Scale. After remission, 15.6% met criteria suggestive of PTSD on the 17-item civilian checklist for PTSD. Stress severity was significantly higher in the acute phase. Severe COVID-19 was associated with cardiovascular comorbidities and diabetes. Individuals with persistent stress or PTSD more frequently relied on disengagement and emotion-focused coping strategies, highlighting a potential behavioral pathway linking pandemic-related stress exposure to increased cardiovascular vulnerability [99].

A large cohort study of SARS-CoV-2-negative ACS patients undergoing PCI demonstrated that the pandemic period was associated with impaired cardiopulmonary recovery despite the absence of viral infection. During the pandemic, peak VO_2_/kg declined by approximately 3–4% compared with pre- and post-pandemic periods, and a higher proportion of patients exhibited abnormal PHQ-9 scores and elevated anxiety levels. Anxiety severity independently predicted poorer cardiopulmonary performance, and lower exercise capacity was reciprocally associated with higher anxiety burden. These findings suggest that pandemic-related psychosocial stress, healthcare avoidance behaviors, reduced physical activity, and delayed care may have contributed to adverse functional recovery patterns in ACS patients [100].

During the COVID-19 pandemic, ACS management required rapid restructuring to balance timely reperfusion with infection control measures. In a single-center registry study, presentations fell by 45%, and the symptom onset–to–first medical contact interval nearly doubled (≈1177 vs. 625 min). Immediate percutaneous coronary intervention was performed less frequently, while thrombolysis and intensified prehospital antiplatelet therapy were used more often, reflecting infection control constraints and time-to-treatment tradeoffs. Despite longer delays and fewer patients achieving door-to-balloon ≤90 min, the composite rate of in-hospital death, cardiogenic shock, sustained ventricular tachycardia or ventricular fibrillation, or mechanical circulatory support was not significantly higher; age remained the main independent predictor of adverse in-hospital outcomes [101].

## 4. Mechanistic Pathways Linking MHD-ACS

The biological mechanisms linking MHDs and ACSs operate bidirectionally. While psychiatric disorders contribute to coronary vulnerability through inflammatory, neuroendocrine, autonomic, platelet, and endothelial pathways, ACSs per se induce systemic inflammation, stress axis activation, autonomic imbalance, and cerebral hemodynamic alterations that increase susceptibility to subsequent depressive and anxiety symptomatology. These reciprocal processes establish a self-reinforcing biological continuum [14,102,103].

To clarify temporal and pathophysiological distinctions, it is important to differentiate mechanisms primarily involved in plaque formation (atherogenesis) from those precipitating plaque destabilization and acute coronary thrombosis. Plaque formation is driven predominantly by chronic endothelial dysfunction [104], lipid accumulation [105], monocyte recruitment [106], and sustained low-grade inflammation that promote gradual intimal thickening and fibrous cap development [107]. In contrast, plaque destabilization involves inflammatory amplification, matrix degradation, endothelial erosion or rupture, platelet activation, and thrombus formation, which together convert a stable lesion into an ACS [108,109]. The following sections distinguish these partially overlapping but pathophysiologically distinct processes.

### 4.1. Chronic Low-Grade Inflammation and Immune Activation

Macrophages detect cellular signals via pattern-recognition receptors (PRRs) and activate nuclear factor kappa B (NF-κB), rapidly inducing pro-inflammatory cytokine release. Classically activated M1 cells upregulate inducible nitric oxide synthase (iNOS) and secrete interleukin-1 beta (IL-1β), interleukin-6 (IL-6), and tumor necrosis factor alpha (TNF-α), sustaining chronic inflammation and tissue injury. In atherosclerosis, excessive M1 polarization promotes plaque progression and instability. In the central nervous system (CNS), oxidative stress drives microglial M1 activation, increasing IL-1β and TNF-α and contributing to neuroinflammation associated with major depressive disorder. During plaque formation, recruited monocyte-derived macrophages internalize native and chemically modified lipoproteins and convert into cholesterol-laden foam cells, driving lesion growth and necrotic core expansion [110]. Uptake of oxidized LDL is mediated in part by scavenger receptors such as CD36, which promotes foam-cell formation in atherosclerotic lesions [111]. In contrast, plaque destabilization reflects a shift toward fibrous-cap weakening: macrophage-derived matrix metalloproteinases degrade cap extracellular matrix, promoting cap thinning and rupture susceptibility in “vulnerable” plaques [112]. Thus, macrophage-driven inflammation represents a shared mechanistic link between cardiovascular plaque destabilization and psychiatric disease [113,114].

Psychological stress disrupts the regulation, biosynthesis, and signaling of noradrenaline, serotonin, dopamine, and cortisol. Such neuroendocrine imbalance promotes upregulation of pro-inflammatory mediators, including IL-1, IL-6, and TNF-α, thereby amplifying systemic inflammation. These pathways are recognized contributors to atherogenesis and increased susceptibility to coronary artery disease [14,115].

Mechanistically, chronic low-grade inflammation is more closely linked to plaque formation, by sustaining endothelial activation and continuous recruitment of circulating monocytes into the arterial wall [107]. In contrast, acute mental stress can preferentially promote plaque destabilization: in atherosclerotic mice, acute stress increased vascular inflammation and accelerated features of plaque vulnerability through sympathetic signaling with norepinephrine-dependent effects on the vessel wall and increased leukocyte recruitment into plaques [116]. In parallel, chronic variable stress activates hematopoietic stem cells via β3-adrenergic signaling in the bone marrow niche, increasing the production of inflammatory leukocytes that can amplify plaque inflammation and vulnerability [117].

Paganin and Signorini indicated that MHDs with prominent inflammatory features exhibit a convergent peripheral and central immune activation signature. This phenotype is defined by broad upregulation of pro-inflammatory cytokines, chemokines, and innate immune markers, accompanied by microglial activation and enhanced toll-like receptor signaling. At the same time, mediators involved in anti-inflammatory regulation and neuroprotection, including IL-4, transforming growth factor beta, and brain-derived neurotrophic factor, are consistently diminished, whereas vascular endothelial growth factor appears variably expressed. Together, these findings delineate a global shift toward sustained innate immune stimulation, chronic neuroinflammation, and compromised neurotrophic resilience across multiple psychiatric disorders [118].

Clinical investigations have demonstrated that individuals exhibiting pronounced depressive symptomatology display markedly elevated circulating levels of -reactive protein (CRP), IL-6, and TNF-α. This inflammatory profile provides further evidence for a strong and clinically meaningful association between immune system activation and depressive pathology [119].

Meta-analytic evidence indicated that heightened systemic inflammation, reflected by increased CRP, hs-CRP, and IL-6 concentrations, is consistently linked to reduced heart rate variability in patients with coronary heart disease and comorbid depression [120]. Another meta-analysis further demonstrated that individuals with depression exhibit significantly higher circulating levels of CRP, IL-6, and TNF-α compared with non-depressed controls [121]. These data suggest that dysregulation of the IL-6/IL-6R signaling axis may contribute to depression vulnerability. Elevated IL-6 is also associated with increased risk of cardiovascular mortality, major adverse cardiovascular events, myocardial infarction, stroke, peripheral arterial disease, and heart failure, highlighting a shared inflammatory pathway linking affective disorders and cardiovascular pathology [122,123].

IL-6 together with IL-1β function as pivotal mediators of sustained, low-intensity inflammation, a pathophysiological state increasingly recognized in both cardiovascular pathology and mental illness. IL-6 is released by immune and vascular cells in response to tissue injury, oxidative stress, and lipid accumulation, and stimulates hepatic CRP synthesis. Besides classical receptor signaling, soluble IL-6R enables IL-6 trans-signaling via gp130 and JAK/STAT activation, prolonging inflammatory gene expression [123,124,125]. IL-6 trans-signaling has been mechanistically implicated in atherogenesis beyond systemic cytokine elevation. In hypercholesterolemic ApoE-/- mice, selective blockade of IL-6 trans-signaling using the sgp130Fc fusion protein significantly reduced atherosclerotic lesion size without altering lipid concentrations, supporting the trans-signaling pathway as a contributor to lesion progression during plaque formation [126]. Translational relevance is supported by CANTOS biomarker analyses, where reduction of cardiovascular events with canakinumab occurred predominantly in patients achieving on-treatment IL-6 levels <1.65 ng/L (HR 0.68, 95% CI 0.56–0.82), independent of LDL lowering [127]. These data link IL-6 pathway activity to clinically measurable coronary risk modulation.

IL-1β is generated mainly by macrophages after NLRP3 inflammasome activation and promotes NF-κB-dependent transcription, leukocyte recruitment, and secondary cytokine release, including IL-6. Together, these interlinked pathways sustain a self-amplifying inflammatory milieu that contributes to shared biological vulnerability in cardiovascular disease and mental illness [123,124,125].

Growing data implicate IL-6-driven immune imbalance as a core biological contributor in schizophrenia. Stress exposure is followed by rapid increases in circulating IL-6, with downstream effects on hematopoiesis, neutrophil trafficking, and CRP production. Either excessive or blunted IL-6 release perturbs Th17 and regulatory T-cell homeostasis, facilitating autoimmune-like responses and persistent low-grade inflammation linked to disease susceptibility. Additionally, IL-6 operates through both canonical receptor-mediated signaling and alternative intracellular pathways. Oxidative stress can modify the lipid and protein organization of the IL-6 receptor complex, favoring non-classical signaling and prolonging maladaptive immune activation. Collectively, these alterations support a central role for disturbed IL-6 signaling within the immunoinflammatory framework of schizophrenia [128].

PTSD is characterized by increased IL-1β signaling, with marked induction under intense stress conditions within the dentate gyrus of the dorsal hippocampus. Stress-related upregulation of IL-1β transcripts and protein expression facilitates maladaptive fear memory formation. In contrast, pharmacological inhibition of IL-1 receptors attenuates PTSD-like phenotypes, supporting a direct mechanistic contribution of central IL-1β pathways to PTSD pathophysiology [129].

TNF-α is a pro-inflammatory cytokine produced by immune and cardiovascular cells in response to ischemia and tissue injury. Its membrane-bound form is cleaved into a soluble active cytokine that signals through TNF receptor type 1 (TNFR1) and TNF receptor type 2 (TNFR2). After myocardial infarction, myocardial TNF-α and receptor expression markedly increase. TNFR1 activation predominantly drives NF-κB-dependent inflammatory and apoptotic pathways, promoting cardiomyocyte death, ventricular remodeling, endothelial activation, leukocyte infiltration, arrhythmias, and contractile dysfunction. By contrast, TNFR2 signaling is associated with partial suppression of inflammation and enhanced angiogenic activity, indicating receptor-specific and dose-dependent effects in cardiovascular disease [130].

Because TNF-α occupies a pivotal position within peripheral and central immune signaling networks, dysregulation of TNF-α-dependent cascades have been consistently linked to the pathophysiology of major psychiatric conditions, including depression [131,132], bipolar disorder [133,134], PTSD [135], anxiety disorders [136], and schizophrenia [137]. This convergent evidence reinforces the concept that immune-driven inflammation constitutes a common biological bridge connecting mental illness with cardiovascular pathology.

C-reactive protein (CRP) has emerged as a clinically relevant indicator of neuroinflammatory activity in psychiatric disease. Elevated circulating CRP concentrations are frequently documented across multiple mental disorders, including schizophrenia, mood disorders, anxiety disorders, and post-traumatic stress disorder. In addition, a state of low-grade systemic inflammation, commonly defined by CRP levels exceeding 3 mg/L, is more often detected in patients with greater symptom severity, poorer therapeutic response, and a more unfavorable illness trajectory. These observations support the concept that an inflammatory subtype of psychiatric illness exists and may warrant distinct clinical stratification and prognostic consideration.

C-reactive protein (CRP) is a neuroinflammatory biomarker with demonstrated roles in psychiatric disorders. CRP is more likely reported to be elevated in several psychiatric disorders, including schizophrenia, mood disorders, anxiety disorders, and post-traumatic stress disorder. Moreover, low-grade inflammation (CRP > 3 mg/L) has been more likely observed in a subgroup of patients affected with a more severe psychopathological symptomatology, more treatment resistance, and worst clinical mental illness course, strengthening the hypothesis of the need for a different clinical and prognostic characterization based on this concomitant neuroinflammatory predisposition [138].

A large systematic review and meta-analysis targeting 13,541 individuals diagnosed with depression and 155,728 non-depressed comparators reported that approximately 25% of depressed patients exhibit markers of low-grade inflammatory activity, while more than 50% display modestly increased CRP concentrations, indicating a substantial inflammatory component in a sizeable proportion of depressive disorders [139]. Several additional investigations indicate that higher baseline CRP concentrations frequently appear before the onset of depressive symptoms, implying that inflammatory activation may act as a predisposing factor in the development of depression rather than representing solely a secondary consequence of the disorder [140,141].

Multiple mechanistic models have been advanced to explain the association between elevated CRP and depressive pathology, yet current evidence suggests that this relationship is shaped by several interacting biological modifiers. CRP exerts isoform-specific biological effects relevant to plaque instability. Native pentameric CRP dissociates into its monomeric form (mCRP) upon interaction with activated platelets in a glycoprotein IIb/IIIa-dependent manner, localizing inflammatory amplification to the atherosclerotic milieu [142,143]. Unlike circulating pentameric CRP, mCRP is detectable within atherosclerotic plaques and promotes complement activation, endothelial adhesion molecule expression, reduced nitric oxide bioavailability, and enhanced platelet aggregation under flow conditions [144]. These mechanisms support a direct role of CRP structural transformation in plaque destabilization and thrombus propagation rather than in early plaque formation.

Inflammatory cytokines together with CRP stimulate indoleamine-2,3-dioxygenase (IDO) [145], the key rate-controlling enzyme that shunts tryptophan toward kynurenine production. As a consequence, less tryptophan remains available for serotonin biosynthesis, resulting in diminished peripheral and central serotonergic tone. Concurrently, activation of the stress axis and sustained hypercortisolemia upregulate tryptophan-2,3-dioxygenase (TDO), amplifying flux through the kynurenine pathway. This metabolic bias favors accumulation of neuroactive and potentially neurotoxic derivatives, most notably quinolinic acid, which perturbs glutamatergic signaling through effects on N-methyl-D-aspartate (NMDA) receptors and contributes to excitatory neurotransmission imbalance [146].

Multiple investigations have identified higher CRP levels and enhanced IDO activation in individuals with major depressive disorder, accompanied by increased kynurenine-to-tryptophan ratios and lower serotonin availability. Collectively, these convergent findings indicate a reciprocal biochemical interplay between inflammatory processes and the pathophysiology of depression [147,148,149]. Concurrently, CRP has emerged as a robust biomarker for estimating cardiovascular risk, operating both as a stand-alone indicator and as a component of multimarker risk models. Growing evidence implicates persistent low-grade inflammatory activity as a fundamental biological driver in the initiation and progression of coronary artery disease [150].

Additionally, fibrinogen, a coagulation-related acute-phase protein with inflammatory properties, shows no reproducible difference between depressed and non-depressed coronary heart disease populations, even in the presence of marked interstudy variability, indicating an unstable link with psychiatric status. In parallel, NT-proBNP, reflecting myocardial strain and neurohormonal activation, does not appear to differ meaningfully between groups. Taken together, these observations point to IL-6 as the most reliable inflammatory correlate of depression in coronary heart disease, whereas fibrinogen and natriuretic peptides exhibit weaker or inconsistent associations [120].

### 4.2. Autonomic Nervous System Dysregulation

Chronic psychological stress alters autonomic equilibrium by persistently activating hypothalamic CRH pathways, thereby favoring sympathetic dominance over parasympathetic control. Projections from the paraventricular nucleus to the locus coeruleus amplify sympathetic tone via α1-adrenergic signaling and concurrently inhibit vagal activity through α2-mediated mechanisms. As a consequence, catecholamine secretion remains chronically elevated, while cholinergic modulation is diminished. Unlike the transient, adaptive autonomic shifts seen in acute stress, prolonged exposure produces sustained sympathetic drive without sufficient parasympathetic opposition. This imbalance manifests clinically as tachycardia, depressed heart rate variability, and reduced cardiovascular resilience, features consistently associated with poor cardiac prognosis [151].

Heart rate variability (HRV) provides a quantifiable marker of autonomic imbalance in depression. A meta-analysis including 2359 depressed patients demonstrated significantly reduced standard deviation of NN intervals (g = −0.87), root mean square of successive differences (g = −0.51), and high-frequency power (g = −0.51) compared with controls, indicating diminished vagal modulation independent of cardiac comorbidity [152].

HRV clinically and non-invasively captures the “signature” of autonomic imbalance relevant to both MHD and coronary risk: chronic stress is associated with sympathetic hyperactivation (↑HR/↑BP, increased hemodynamic load) and vagal withdrawal (↓parasympathetic modulation). The vagus nerve exerts anti-inflammatory effects through the cholinergic anti-inflammatory pathway, limiting macrophage activation and cytokine release. When vagal tone declines (reduced HRV), this anti-inflammatory restraint weakens, promoting a pro-inflammatory milieu (including inverse correlations between HRV and markers such as IL-6 and CRP) that accelerates inflammaging, plaque instability, and vulnerability to ACS events [153].

In post-myocardial infarction patients with major depression, impaired HRV recovery persisted over follow-up, whereas improvement in depressive symptoms was accompanied by partial HRV normalization [154]. These data support reduced vagal tone as a measurable physiological intermediary linking depression to adverse cardiac prognosis.

Across the spectrum of CVDs, persistent amplification of sympathetic tone constitutes a fundamental pathophysiological force driving both onset and disease worsening. Evidence from microneurography and norepinephrine kinetic studies reveals progressively heightened sympathetic output in individuals with high-normal blood pressure, overt hypertension, and treatment-resistant hypertension, closely tracking the magnitude of blood pressure elevation. Such neuroadrenergic excess facilitates hypertension-related target organ injury, manifested by endothelial impairment, vascular stiffening, and concentric myocardial hypertrophy. Within cardiac tissue, prolonged β1-adrenergic activation enhances cytosolic Ca^2+^ fluxes, transiently increasing inotropy while simultaneously raising myocardial oxygen demand, a combination that accelerates maladaptive remodeling. In parallel, augmented sympathetic drive stimulates the renin–angiotensin–aldosterone axis, amplifying preload and afterload and sustaining a self-perpetuating cycle of ventricular dysfunction. Collectively, these neurohumoral and structural perturbations increase susceptibility to heart failure, major adverse cardiac events, and ultimately ACS, underscoring sympathetic hyperactivation as a key biological link between autonomic imbalance and cardiovascular risk [155].

During the acute phase of ACS, heightened sympathetic drive and increased circulating inflammatory markers rise in parallel and subsequently diminish with recovery, suggesting coexisting but independent biological pathways that each contribute to unfavorable cardiovascular prognosis [156].

### 4.3. HPA Axis Hyperactivation

Neuroendocrine stress regulation is increasingly implicated in the pathophysiology of multiple chronic disorders, with the hypothalamic–pituitary–adrenal (HPA) axis occupying a pivotal position in this process [157]. Functionally, the HPA axis integrates hypothalamic, pituitary, and adrenal signaling into a coordinated neuroendocrine network that orchestrates adaptive responses to environmental and internal challenges, thereby preserving physiological equilibrium [158]. Converging clinical and experimental data indicate that this system is persistently overactivated in individuals with depressive disorders when compared with non-affected populations [158,159].

Complex neuromodulatory networks exert continuous control over the HPA axis, and these regulatory systems are profoundly disrupted across psychiatric illnesses. Exposure to stress engages serotonergic, noradrenergic, and dopaminergic projections that converge on corticotropin-releasing, hormone-producing neurons within the paraventricular nucleus, leading to enhanced corticotropin-releasing hormone gene expression and peptide release. Activation of 5-HT1A and 5-HT2 receptors by serotonin, stimulation of α1-adrenergic receptors by norepinephrine, and signaling through dopamine D1 receptors collectively facilitate adrenocorticotropic hormone secretion and raise systemic cortisol concentrations. In parallel, increased excitatory glutamatergic drive toward paraventricular nucleus corticotropin-releasing hormone neurons augments adrenocorticotropic hormone and corticosterone output, whereas attenuation of γ-aminobutyric acid-mediated tone removes an important inhibitory brake on HPA axis activity. Neuropathological investigations in mood disorders reveal expanded populations of corticotropin-releasing hormone-positive and corticotropin-releasing hormone/arginine vasopressin-coexpressing neurons, higher corticotropin-releasing hormone messenger ribonucleic acid (mRNA) expression, and elevated circulating corticotropin-releasing hormone levels [160].

Alterations of the HPA axis may present as reduced basal cortisol output, blunted or exaggerated stress reactivity, or loss of normal diurnal cortisol rhythmicity. Prolonged exposure to stress typically produces an early phase of sustained hypercortisolemia, followed by receptor downregulation and progressive signaling exhaustion, thereby aggravating neuroendocrine dysregulation. This maladaptive trajectory is associated with heightened vulnerability to cardiovascular, metabolic, immune, and psychiatric disorders, as well as cognitive decline, and may also contribute to mechanisms involved in neurodegenerative disease [157].

Persistent HPA axis overdrive produces chronic cortisol excess that weakens glucocorticoid receptor feedback, sustaining corticotropin-releasing hormone–adrenocorticotropic hormone signaling and long-term hypercortisolemia. In depressive and bipolar disorders, this state disrupts circadian rhythms, reduces receptor sensitivity, and suppresses brain-derived neurotrophic factor, thereby impairing neuroplasticity. Cortisol simultaneously diminishes serotonergic tone by increasing serotonin transporter activity, lowering tryptophan availability, and modifying 5-HT2C receptor function. Prolonged exposure promotes hippocampal atrophy, reduced neurogenesis, and cognitive-affective dysfunction. In psychotic and anxiety disorders, elevated cortisol compromises hippocampal–prefrontal circuits, enhances neuroinflammatory processes, and correlates with symptom severity, while glucocorticoid resistance blunts anti-inflammatory actions. Therefore, cortisol dysregulation constitutes a shared endocrine pathway contributing to multiple psychiatric conditions [161,162].

Conversely, elevated concentrations of circulating stress hormones were strongly correlated with a greater likelihood of developing cardiovascular pathology when contrasted with lower hormonal levels. Overall risk modeling demonstrated an approximate 63% increase in cardiovascular risk among individuals exhibiting globally higher stress hormone profiles. When evaluated separately, excess norepinephrine was associated with an estimated 68% rise in cardiovascular risk, increased epinephrine with a 58% higher risk, and elevated cortisol with an approximate 60% greater risk, underscoring a robust dose–risk relationship between neuroendocrine activation and cardiovascular vulnerability [163].

### 4.4. Platelet Hyperreactivity

While inflammatory and endothelial mechanisms contribute primarily to plaque development, platelet activation is central to the transition from plaque destabilization to occlusive thrombosis [164].

Platelets form an important biological bridge between psychiatric disorders and thrombotic cardiovascular risk because of their close functional similarity to central monoaminergic systems [165]. Structural and functional disturbances in platelet morphology and signaling, influencing neurotransmitter release, calcium homeostasis, and inflammatory mediator secretion, are evident across multiple disorders. These alterations favor systemic inflammation, oxidative stress, and blood–brain barrier disruption, with emerging evidence implicating modulation by the gut microbiota [166].

Heightened platelet reactivity has been proposed as a contributory biological mechanism underlying the greater susceptibility of individuals with depression to the development of ischemic heart disease [167]. In depression, disrupted serotonergic and noradrenergic platelet signaling alters serotonin storage and uptake, lowering activation thresholds and producing a hyperreactive, prothrombotic phenotype. In schizophrenia, structural and metabolic platelet abnormalities, together with oxidative stress, modify aggregation responses. Anxiety and suicidality are likewise associated with serotonergic dysfunction and reduced inhibitory receptor density on platelets. Collectively, these alterations converge toward increased platelet reactivity, promoting thrombosis, plaque destabilization, and greater susceptibility to ACS [165].

During ACSs, platelets act as key mediators of arterial thrombosis after disruption of an atherosclerotic plaque. Contact with exposed collagen and von Willebrand factor triggers platelet tethering and adhesion through surface receptors such as GPIb-IX-V, GPVI, and α2β1, promoting rapid recruitment and heightened responsiveness. Downstream activation of intraplatelet signaling and thrombo-inflammatory pathways strengthens aggregation and supports growth of an occlusive thrombus, limiting coronary perfusion and precipitating myocardial ischemia. At the same time, these pathways integrate hemostatic and inflammatory signaling, highlighting a close interplay between prothrombotic and pro-inflammatory platelet states in acute coronary syndromes [168].

### 4.5. Endothelial Dysfunction

Within ACS, impaired endothelial function emerges as an independent prognostic determinant, being associated with an approximately 2.4-fold increase in the risk of cardiovascular mortality. This observation supports a direct connection between antecedent endothelial damage and unfavorable outcomes during acute coronary events [169].

Depressive illness has been consistently linked to abnormalities in vascular homeostasis, manifested by blunted endothelium-dependent vasorelaxation that reflects compromised nitric oxide (NO) signaling. Heightened inflammatory burden, characterized by elevated cytokines, adhesion molecules, and acute-phase reactants, interferes with endothelial function and diminishes NO bioavailability. While high-density lipoprotein typically supports endothelial regeneration and NO production, these atheroprotective actions appear weakened in individuals with depression. In contrast, vasodilatory responses that bypass the endothelium remain intact, indicating preserved smooth muscle reactivity. This profile suggests an early stage of endothelial injury and provides a plausible biological pathway connecting affective disorders with heightened cardiovascular vulnerability [170].

Concurrently, prolonged hyperactivity of the HPA axis in psychiatric conditions results in continuous cortisol excess and progressive insensitivity of endothelial glucocorticoid receptors. This alteration weakens the physiological anti-inflammatory effects of glucocorticoids, allowing cytokine-driven signaling to persist within the endothelium and further impair nitric oxide-mediated vasoregulatory mechanisms. Ineffective glucocorticoid feedback additionally promotes increased expression of endothelial adhesion molecules and destabilization of barrier integrity, thereby enhancing leukocyte adhesion and vascular inflammatory responses. Together, glucocorticoid receptor resistance and ongoing inflammatory activation cooperate to sustain diminished NO availability and endothelial dysfunction, consolidating the heightened vascular risk characteristic of psychiatric illness [171].

Chronic glucocorticoid excess directly impairs endothelial nitric oxide signaling. In cultured human endothelial cells, dexamethasone suppresses endothelial nitric oxide synthase (eNOS) transcription and reduces NO production, effects mediated via glucocorticoid receptor-dependent pathways [172]. In patients with coronary artery disease, acute mental stress significantly reduces flow-mediated dilation, an effect prevented by pharmacologic inhibition of cortisol synthesis with metyrapone. These findings define a clinically demonstrable HPA-axis–cortisol–eNOS suppression cascade linking psychiatric stress biology to impaired vascular reactivity and coronary vulnerability [173].

Converging evidence indicates that mental health-related neuroendocrine and inflammatory dysregulation promotes endothelial dysfunction, which, in turn, contributes to both progressive atherogenesis and increased susceptibility to plaque destabilization, establishing a bidirectional ACS–mental health continuum.

Across these pathways, chronic immune activation predominantly drives plaque initiation and growth, whereas sympathetic surges, protease activity, and thrombo-inflammation more directly govern cap failure and thrombosis, the proximate events in ACS.

Table 2 synthesizes the principal biological pathways through which MHDs and ACSs converge, emphasizing shared mediators and bidirectional pathophysiological mechanisms.

**Figure 3:**
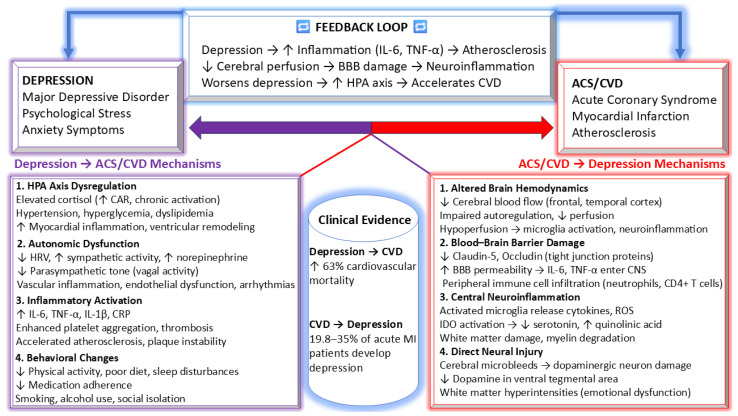
Integrated pathophysiological mechanisms underlying the bidirectional relationship between ACS/CVD and depression. Conceptual framework depicting the bidirectional pathophysiological links between depression and ACS/CVD. The left panel summarizes mechanisms by which depression promotes adverse cardiovascular outcomes (neuroendocrine dysregulation, autonomic imbalance, inflammatory activation, and maladaptive behaviors), whereas the right panel illustrates pathways through which ACS/CVD contributes to the development of depressive symptoms (altered cerebral perfusion, blood–brain barrier disruption, central neuroinflammation, and direct neural injury). The central feedback loop emphasizes the dynamic amplification between the two disease processes. ACS, acute coronary syndrome; CVD, cardiovascular disease; HPA axis, hypothalamic–pituitary–adrenal axis; CAR, cortisol awakening response; HRV, heart rate variability; IL-6, interleukin-6; TNF-α, tumor necrosis factor alpha; IL-1β, interleukin-1 beta; CRP, C-reactive protein; BBB, blood–brain barrier; CNS, central nervous system; ROS, reactive oxygen species; IDO, indoleamine 2,3-dioxygenase; MI, myocardial infarction; ↑ increased levels or activity; *↓* decreased levels or activity.

Overall, these inter-related inflammatory, neuroendocrine, autonomic, platelet, and endothelial pathways establish a self-reinforcing biological network linking mental health disorders and acute coronary syndrome within a bidirectional disease continuum. The bidirectional relationship between depression and ACS/CVD operates through multiple interconnected pathways (Figure 3). Depression promotes cardiovascular disease via HPA axis dysregulation, autonomic dysfunction, inflammatory activation, and behavioral modifications. Conversely, ACS/CVD triggers depression through altered cerebral hemodynamics, blood–brain barrier disruption, neuroinflammation, and direct neural damage. These mechanisms create a self-perpetuating feedback loop where each condition amplifies the other, supported by substantial clinical evidence showing increased mortality and morbidity in comorbid patients [174].

The converging evidence supports the conceptualization of depression and anxiety as prototypical and clinically prevalent expressions of broader neuroinflammatory, neuroendocrine, autonomic, platelet, and endothelial dysregulation that characterize the mental health–ACS interface. Moreover, these shared biological pathways extend across the wider spectrum of mental health disorders and operate bidirectionally, as acute coronary syndromes themselves induce systemic inflammation, stress axis activation, cerebral perfusion alterations, and blood–brain barrier disruption that may precipitate or exacerbate affective symptomatology. Together, these mechanisms reinforce a unified and self-perpetuating mechanistic continuum rather than a linear cause–effect model.

### 4.6. Pharmacological Modulation of Shared Inflammatory Pathways

#### 4.6.1. Modulation of Shared Pathways by Psychiatric Pharmacotherapy

Selective serotonin reuptake inhibitors (SSRIs) actively modulate the inflammatory, endothelial, platelet, and autonomic pathways. Regarding vascular inflammation, sertraline reduced circulating CRP and IL-6 in coronary heart disease patients with depression. SSRI-associated reductions in IL-1β, a key NLRP3-driven mediator of both plaque progression and neuroinflammation, have been observed. Concerning endothelial function, escitalopram decreased VCAM-1 and von Willebrand factor, while paroxetine increased serum nitric oxide and eNOS activity through a direct, depression-independent mechanism, partially reversing the endothelial NO deficiency. Platelet hyperreactivity was attenuated by sertraline, which reduced beta-thromboglobulin, P-selectin, PF-4, and thromboxane B2 in post-ACS patients despite concurrent antiplatelet therapy. Regarding autonomic pathways, SSRIs reduced arterial stiffness and augmentation index exclusively in treatment responders, suggesting that vascular benefit is partly mediated through depression remission rather than direct pharmacological action alone [175].

Antidepressant classes exhibit differential electrophysiologic and autonomic profiles that may modify cardiovascular risk. Tricyclic antidepressants inhibit cardiac fast sodium channels and have anticholinergic/adrenergic effects that prolong PR/QRS/QT intervals, reduce heart rate variability, and increase arrhythmia risk, mechanisms that can worsen autonomic imbalance and electrical instability in CAD. In contrast, SSRIs are generally better tolerated at therapeutic doses and may attenuate thrombotic risk by inhibiting serotonin uptake into platelets. Sertraline appears neutral or modestly favorable regarding heart rate variability; however, QT prolongation can occur in susceptible individuals, particularly with higher doses or drug interactions, warranting electrocardiographic monitoring [176].

Pharmacovigilance analyses have identified disproportionate reporting signals linking SSRIs to arrhythmias, QT prolongation, and rare cardiomyopathy events. These observational signals do not establish causality but are consistent with known electrophysiologic mechanisms, including blockade of the cardiac rapid delayed rectifier potassium channel encoded by the human ether-à-go-go-related gene, which delays ventricular repolarization. These findings support careful risk stratification and monitoring in high-risk populations rather than routine avoidance [177].

Antipsychotics modify the same autonomic, electrophysiologic, metabolic, and inflammatory pathways central to ACS vulnerability. Alpha-1 adrenergic blockade contributes to orthostatic hypotension, whereas muscarinic antagonism and catecholamine elevation may promote persistent tachycardia and sympathetic predominance. Most agents inhibit cardiac repolarizing potassium currents, predisposing to QT prolongation and malignant arrhythmias. Clozapine-induced myocarditis involves catecholaminergic activation, cytokine release, and IgE-mediated inflammation, directly amplifying the pro-inflammatory pathways. Conversely, 5-HT2A receptor blockade by atypical antipsychotics may partially attenuate platelet aggregation, potentially moderating thrombotic risk. Second-generation antipsychotics may additionally promote dyslipidemia and insulin resistance, indirectly amplifying atherosclerotic substrate formation. Collectively, these mechanisms demonstrate that psychiatric pharmacotherapy does not merely coexist with cardiovascular risk but actively interacts with the inflammatory and autonomic circuits described above [178,179,180].

#### 4.6.2. Anti-Inflammatory Modulation of Shared Pathways

Emerging evidence positions vascular inflammation not merely as a consequence of atherosclerosis but as a primary, modifiable driver of residual cardiovascular risk, including in post-percutaneous coronary intervention patients, independent of lipid control [105]. A pooled analysis of STRENGTH, REDUCE-IT, and PROMINENT demonstrated that hsCRP predicted cardiovascular events more strongly than LDL-C in statin-treated patients [181].

The CANTOS trial confirmed this paradigm, showing that canakinumab, an IL-1β inhibitor, at 150 mg every three months significantly reduced recurrent cardiovascular events independently of lipid lowering, with concomitant reductions in hsCRP and IL-6 [182].

Colchicine, acting via tubulin disruption and NLRP3 inflammasome inhibition, reduced ischemic events by 23% in post-MI patients in the COLCOT trial. Moreover, it has been demonstrated that colchicine significantly increased fibrous cap thickness, directly attenuating plaque vulnerability [183,184].

Ziltivekimab, targeting IL-6, markedly reduced hsCRP by up to 92% at 30 mg in high-risk patients. Given that shared inflammatory pathways, particularly IL-1β, IL-6, and NLRP3, underlie both atherosclerotic plaque destabilization and neuroinflammation implicated in mental disorders onset, anti-inflammatory strategies may represent a converging therapeutic target at the MHD-ACS interface [185].

**Figure 4:**
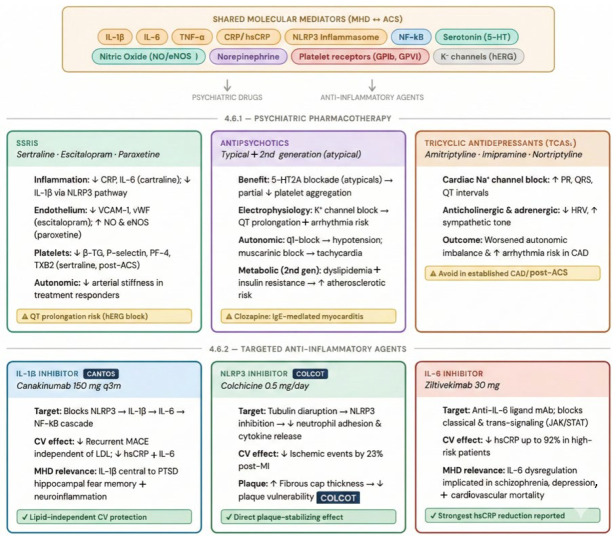
Pharmacological modulation of shared pathways at the MHD–ACS interface. The upper panel identifies key shared molecular mediators linking MHD and ACS. The lower panels illustrate how psychiatric pharmacotherapy (SSRIs, antipsychotics, and tricyclic antidepressants) and targeted anti-inflammatory agents (canakinumab, colchicine, and ziltivekimab) interact with these convergent pathways. Green benefit tags indicate cardioprotective effects; yellow warning tags denote adverse cardiovascular signals requiring monitoring; ↑ increased levels or activity; ↓ decreased levels or activity.

The pharmacological interactions between psychiatric medications and the shared inflammatory, autonomic, platelet, and endothelial pathways implicated in both MHD and ACS are summarized in Figure 4 [105,175,176,177,178,179,180,181,182,183,184,185].

## 5. Interventions in MHD-ACS Relationship

The reciprocal interplay between psychiatric morbidity and ACS necessitates a multidimensional and coordinated management strategy. Effective care should combine pharmacotherapy with evidence-based psychological interventions, structured collaborative care frameworks, and close integration within cardiac rehabilitation programs, thereby addressing both mental health and cardiovascular risk in a unified therapeutic model [186].

### 5.1. Pharmacological Treatment

In patients with MHDs, platelet hyperreactivity may plausibly contribute to a higher thrombotic risk during ACS. While no ACS risk score or antithrombotic strategy is currently tailored to psychiatric status, escalation to parenteral antiplatelet therapy may be considered in selected high-risk scenarios. Cangrelor, an intravenous, rapidly acting, and reversible P2Y12 inhibitor, provides immediate platelet inhibition during percutaneous coronary intervention and has shown lower periprocedural ischemic complications compared with oral P2Y12 [187].

Glycoprotein IIb/IIIa inhibitors (e.g., tirofiban) remain guideline indicated mainly as bail-out therapy (e.g., large thrombus/no-reflow) but require careful bleeding risk assessment [188]. However, tirofiban may represent a potent parenteral bridge for patients with high thrombotic burden and neuroendocrine-driven procoagulant activity [189].

Data derived from real-world cohorts consistently support selective serotonin reuptake inhibitors as the pharmacological class with the strongest evidence base for managing depression in individuals with coronary artery disease or ACS. Within this population, SSRIs demonstrate a favorable safety profile and clinically meaningful antidepressant efficacy. Among available agents, sertraline has been investigated most extensively and is often considered a preferred option, largely because it lacks clinically relevant QT-interval prolongation and exhibits good cardiovascular tolerability [190].

Compared with SSRIs, serotonin–norepinephrine reuptake inhibitors (SNRIs) enhance both serotonergic and noradrenergic transmission and may therefore lead to modest elevations in heart rate and arterial pressure, warranting careful hemodynamic surveillance, particularly in patients receiving venlafaxine. Tricyclic antidepressants (TCAs) and monoamine oxidase inhibitors (MAOIs) exhibit substantial cardiovascular liability, including impaired cardiac conduction, proarrhythmic effects, orthostatic hypotension, and risk of hypertensive crises, which largely precludes their use in individuals with established CVD. In contrast, atypical agents such as mirtazapine and trazodone generally demonstrate a more favorable cardiac safety profile, although dose-related hypotension and occasional QT-interval prolongation have been reported [176].

A quantitative synthesis examining cardiovascular outcomes in depressed individuals with coronary artery disease demonstrated that exposure to SSRIs after ACS is linked to a substantial decrease in recurrent ischemic events. Specifically, patients receiving SSRI therapy in the post-ACS setting exhibited an approximate 44% relative reduction in the risk of subsequent myocardial infarction, supporting a potential cardioprotective effect of these agents in this high-risk population [191].

Randomized placebo-controlled investigations, including CREATE [192], EsDEPACS [193,194], and SADHART [195], consistently show that sertraline, escitalopram, and citalopram provide greater improvement in depressive symptoms than placebo in patients with coronary artery disease or ACS. Extended observation from EsDEPACS additionally indicates that escitalopram therapy is associated with fewer major adverse cardiac events and reduced all-cause mortality across long-term follow-up. Although most randomized trials were designed primarily for psychiatric outcomes rather than cardiovascular endpoints, real-world observational studies report lower mortality among patients treated with SSRIs and among those achieving antidepressant response. By comparison, antidepressant therapy demonstrates modest benefit in heart failure populations, yet SSRIs appear to have an acceptable safety profile. Tricyclic antidepressants are generally avoided because of well-established cardiotoxic effects, while data supporting other antidepressant classes in cardiac populations remain limited.

Evidence that depression treatment reduces recurrent ischemic events after ACS remains mixed. In ENRICHD, cognitive behavioral therapy with or without antidepressants improved psychosocial outcomes but did not significantly reduce the primary composite of death or recurrent myocardial infarction compared with usual care. In contrast, the COPES randomized trial of a patient-preference, stepped-care depression program after ACS reported fewer major adverse cardiac events versus usual care, although cardiovascular outcomes were secondary and the trial was not powered primarily for hard endpoints [190].

The clinical impact of treating depression on recurrent ACS remains a subject of intense investigation. Evidence from the K-DEPACS trial demonstrated that successful antidepressant treatment with escitalopram significantly reduced the 8-year risk of MACEs compared to placebo [196].

Within the UPBEAT study, sertraline achieved a significantly larger decrease in depressive symptom burden than placebo, with overall antidepressant efficacy similar to that observed with structured aerobic training. Nonetheless, treatment with sertraline was accompanied by higher rates of fatigue and sexual adverse effects relative to both exercise and placebo. From a physiological perspective, sertraline produced a small enhancement in baroreflex sensitivity when compared with exercise, yet it did not demonstrate reproducible improvements in indices of chronic inflammation, endothelial performance, or platelet reactivity. Taken together, these data reinforce SSRIs, and sertraline in particular, as effective and broadly safe agents for managing depression in coronary heart disease while indicating that convincing evidence for intrinsic cardioprotective actions remains limited [197].

Among individuals who develop depressive syndromes following an ACS, administration of escitalopram at doses of 5–20 mg daily over a 24-week period has been shown to provide robust antidepressant benefit while maintaining a favorable cardiovascular safety profile. Within a randomized, double-blind, placebo-controlled design, escitalopram produced a significantly greater decline in Hamilton Depression Rating Scale scores and yielded parallel improvements on the Montgomery–Åsberg depression rating scale, Beck depression inventory, clinical global impression-severity scale, and measures of social and occupational functioning. The treatment was not accompanied by detrimental effects on echocardiographic or electrocardiographic indices, arterial pressure, body mass, or routine laboratory markers. Collectively, these data position escitalopram as a well-tolerated and evidence-supported pharmacologic option at the interface of depressive pathology and CVD [198].

Antidepressant safety is class dependent. Tricyclic antidepressants exhibit significant cardiotoxicity by inhibiting fast sodium channels, slowing conduction velocity (prolonging PR, QRS, and QT intervals), and increasing risks for AV block and reentry arrhythmias. Conversely, SSRIs (e.g., sertraline and fluoxetine) are considered first line for ACS patients due to their acceptable safety profile and potential cardioprotective antiplatelet effects. However, citalopram demonstrates dose-dependent cardiotoxicity, particularly QTc prolongation. SNRIs (e.g., venlafaxine) may increase heart rate and blood pressure via norepinephrine reuptake inhibition, while atypical agents like mirtazapine and trazodone generally show minimal cardiovascular impact at therapeutic doses [176].

Pharmacological treatment of BD in patients with CVD prioritizes mood stabilizers and antipsychotics with favorable cardiac safety. Lithium may produce repolarization changes, atrioventricular block and sinus bradycardia. Valproate mainly affects platelet function without major cardiotoxicity, whereas carbamazepine can impair cardiac conduction. Lamotrigine and oxcarbazepine show minimal cardiovascular risk. Typical antipsychotics are strongly associated with QT/QTc prolongation and Torsades de pointes. Among atypical agents, clozapine, risperidone, and quetiapine commonly cause orthostatic hypotension and metabolic abnormalities, while aripiprazole has the safest cardiac profile. Antipsychotics remain the cornerstone of schizophrenia management, with routine monitoring of metabolic and cardiac parameters. Antipsychotics may provide heterogeneous cardiovascular risks. Most clinical concern involves dose-dependent QTc prolongation via blockade of the delayed rectifier potassium current, which increases susceptibility to Torsades de Pointes and sudden cardiac death. Thioridazine and haloperidol exhibit higher torsadogenic potential, whereas aripiprazole and lurasidone show minimal QTc impact. Beyond arrhythmias, antipsychotics may cause orthostatic hypotension and sinus tachycardia through alpha1-adrenergic and muscarinic M2 blockade, respectively. Critically, clozapine is uniquely associated with life-threatening myocarditis and cardiomyopathy, necessitating monitoring for tachycardia and flu-like symptoms during treatment initiation [199].

Clozapine carries additional risk of myocarditis, cardiomyopathy, and rare sudden cardiac death. For anxiety disorders, first-line pharmacotherapy includes SSRIs and serotonin–norepinephrine reuptake inhibitors (SNRIs). Benzodiazepines may be used short-term, preferably short- or intermediate-acting agents such as lorazepam or oxazepam, with negligible cardiovascular effects. Quetiapine or gabapentin may serve as second-line options [200].

Growing evidence indicates that traditional Chinese medicine contributes to modulation of the MHD-CVD interface through individualized, pattern-oriented, multi-pathway interventions. Clinical data suggest that Xinkeshu administration following percutaneous coronary intervention is associated with improvement in depressive symptom burden together with favorable changes in lipid metabolism. Quantitative syntheses report better outcomes compared with absence of therapy and therapeutic performance comparable to conventional pharmacological approaches. At the molecular level, phytochemicals including quercetin, kaempferol, puerarin, baicalein, and tanshinone IIA display anti-inflammatory, antioxidative, and cytoprotective actions [201].

Several nonconventional pharmacologic and nutraceutical strategies have been explored for depressive symptom management. *Hypericum perforatum* demonstrates superiority over placebo and therapeutic effects comparable to standard antidepressants in mild, non-melancholic depression. S-adenosyl-L-methionine (SAM-e) shows possible benefit in depression, although available evidence remains of modest methodological strength. Long-chain omega-3 polyunsaturated fatty acids, particularly eicosapentaenoic acid and docosahexaenoic acid, are associated with a reduced likelihood of depressive symptoms in meta-analytic evaluations. Importantly, robust data regarding the safety and efficacy of these interventions in coronary artery diseases populations are lacking, highlighting the need for dedicated cardiovascular-focused trials [202].

### 5.2. Psychotherapy and Stress-Reduction Interventions

Among psychotherapeutic interventions, cognitive behavioral therapy (CBT) has the strongest evidence base for treating depression in individuals with CVD. This modality targets maladaptive thought patterns, dysfunctional behaviors, and impaired emotional regulation. Structured telephone-based CBT interventions, such as the MoodCare program, deliver approximately ten clinician-guided sessions focused on psychological and cardiovascular risk reduction and have been shown to significantly alleviate depressive symptoms following ACS. Likewise, web-based CBT platforms administered over nine to twelve weeks, including nurse-supported models, are associated with meaningful improvements in depression severity [190].

Within the ENRICHD study, individuals treated after myocardial infarction with CBT demonstrated larger improvements in depressive symptom severity, as reflected by greater decreases in HAM-D scores, relative to participants assigned to educational support. However, no significant differences were observed between groups for primary cardiovascular endpoints. Notably, subsequent exploratory analyses indicated that engagement in physical exercise correlated with lower rates of all-cause mortality and recurrent non-fatal MI [203,204].

Additional structured psychotherapeutic modalities, including interpersonal-focused and problem-oriented therapies, have demonstrated efficacy in alleviating depressive symptomatology, enhancing adherence to medical regimens, and lowering relapse vulnerability when used as adjuncts to pharmacotherapy. These interventions typically yield progressive symptomatic benefit and measurable gains in health-related quality of life. While consistent reductions in cardiac-specific mortality have not been established, the integration of psychological care with routine cardiologic management is linked to fewer fatal cardiac events and superior mental health outcomes relative to usual care alone, without a parallel effect on overall survival [201].

Among individuals with CVD complicated by anxiety disorders, structured psychotherapeutic strategies yield moderate but clinically relevant symptom improvement. CBT represents the most extensively evaluated modality, while growing evidence also supports mindfulness-oriented therapies, metacognitive approaches, eye movement desensitization and reprocessing, behavioral activation, relaxation-integrated CBT, stress-reduction programs, and social–cognitive interventions. Programs explicitly designed to address anxiety demonstrate superior efficacy compared with nonspecific psychological interventions [205].

### 5.3. Multidisciplinary and Stepped-Care Models

Available data support the implementation of an integrated, multi-component care model that incorporates psychological therapy, appropriate pharmacologic treatment, structured physical activity, stress-reduction strategies, counseling modalities, and patient-centered self-care interventions. Systematic screening for depressive symptoms, together with coordinated multidisciplinary management, is essential for maximizing psychiatric stabilization and improving cardiovascular prognosis in this high-risk population. Contemporary collaborative care frameworks prioritize systematic identification of depressive symptomatology in individuals with coronary heart disease, with subsequent escalation of integrated therapeutic strategies according to clinical burden and psychosocial needs. Empirical findings indicate that coupling detection procedures with structured self-care guidance for mood symptoms yields measurable improvements in patient outcomes [201].

Guidelines recommendations endorse comprehensive care models that merge behavioral stress reduction, counseling formats, patient-directed self-management, adjunctive pharmacotherapy, and psychotherapeutic interventions within collaborative treatment frameworks. In individuals presenting with moderate-to-severe depressive symptomatology, parallel implementation of psychotherapy, antidepressant medication, and structured longitudinal monitoring demonstrates superior efficacy compared with isolated strategies. Cardiology providers are advised to incorporate routine mood screening into clinical practice, whereas psychiatric specialists should consider cardiovascular safety when selecting antidepressant agents. In complex cases, coordinated multidisciplinary teams integrating cardiology, psychiatry, and psychology formulate personalized management plans that simultaneously target cardiac pathology and mental illness, with the overarching objective of enhancing functional status and long-term prognosis despite persistent gaps in standardized guidance [190,201]. Tiered treatment frameworks in cardiovascular cohorts rely on repeated evaluation of psychological status and the progressive intensification of psychotherapeutic and/or pharmacologic interventions based on symptom burden and therapeutic response [190].

Among individuals hospitalized for ACS who exhibit clinically significant depressive symptomatology, the COPES study demonstrated that a six-month, preference-driven, remotely delivered stepped-care program achieved superior improvement in depressive severity, as reflected by BDI scores, and was associated with fewer major adverse cardiovascular events compared with standard management [206].

Consistent with these findings, the CODIACS study reported meaningful reductions in depressive burden at six months when stepped-care interventions were delivered through telephone- or web-based platforms following ACS [207]. By contrast, the expanded CODIACS-QoL strategy, which integrated systematic depression screening with stepped-care management, failed to demonstrate advantages in depression-free survival, symptom severity, quality-adjusted life expectancy, or mortality over screening with provider feedback or usual care. Taken together, stepped-care frameworks offer a structured, patient-tailored method for escalating treatment that consistently alleviates depressive symptoms, while effects on cardiovascular endpoints remain variable [208].

#### 5.3.1. Structured Screening and Diagnostic Pathways

The Patient Health Questionnaire-9 (PHQ-9) is a nine-item self-report instrument assessing the frequency of depressive symptoms over the preceding two weeks, with each item scored 0–3, yielding a total range of 0–27. It evaluates core Diagnostic and Statistical Manual of Mental Disorders (DSM)-based criteria encompassing both somatic-affective dimensions (fatigue, sleep disturbance, appetite changes, and psychomotor alterations) and cognitive-affective dimensions (anhedonia, depressed mood, concentration difficulties, feelings of worthlessness, and suicidal ideation). It functions as a screening and severity stratification tool, not a diagnostic instrument; elevated scores indicate symptom burden consistent with depression but require confirmatory clinical evaluation. Serial administration enables objective treatment response monitoring, with a ≥5-point score reduction representing a clinically meaningful improvement threshold [209,210]. The PHQ-9 can be conducted after hospital discharge or within 2–3 months following ACS, with repeat assessment throughout cardiac rehabilitation [209].

A large individual participant data meta-analysis encompassing 44,503 participants across 100 studies established that at the standard cutoff of ≥10, PHQ-9 sensitivity and specificity were both 0.85 against semistructured diagnostic interviews, which most closely replicate clinical diagnostic procedures. When restricted exclusively to participants not currently diagnosed or receiving mental health treatment, the population that would actually undergo screening in practice, specificity improved to 0.89 while sensitivity remained unchanged. The area under the curve reached 0.90 against semistructured reference standards. Importantly, positive predictive values ranged from only 23% to 65% depending on population prevalence, underscoring that positive screens require confirmatory clinical evaluation rather than direct treatment initiation [211].

Based on the Yuan et al. study of 782 Chinese ACS inpatients validated against the Mini International Neuropsychiatric Interview, the PHQ-9 demonstrated superior screening performance compared with Hospital Anxiety and Depression Scale–Depression Subscale (HADS-D). At the optimal cutoff of ≥10, PHQ-9 achieved sensitivity of 86.9%, specificity of 84.7%, and a negative predictive value of 97.2%, with an AUC of 0.842, indicating moderate-to-good diagnostic accuracy. Sensitivity was significantly higher than HADS-D (76.2%, *p* = 0.001), yielding fewer missed diagnoses. This advantage reflects PHQ-9’s dual somatic-affective and cognitive-affective coverage, whereas HADS-D captures only cognitive-affective symptoms, omitting somatic criteria central to DSM-based major depressive disorder diagnosis in ACS populations [34].

In a randomized controlled trial of 200 ACS patients with mild-to-moderate depression, PHQ-9 was administered 1–8 weeks post-myocardial infarction as the primary screening instrument, consistent with AHA guidance recommending systematic depression assessment within 2–3 months of ACS onset. Serial PHQ-9 measurements at 6, 12, and 18 months demonstrated progressively stronger negative correlations with quality-of-life scores, confirming its longitudinal validity as both a screening and treatment-monitoring instrument in post-ACS populations [212].

Generalized anxiety disorder-7 (GAD-7) is a seven-item self-report scale assessing generalized anxiety symptoms over a two-week period, with a total score range of 0–21. Because anxiety frequently coexists with depression after ACS and independently predicts poorer adherence and health behaviors, routine anxiety screening should accompany depression assessment [213].

In a validation study of 398 coronary heart disease inpatients, GAD-7 demonstrated robust psychometric properties specifically within a cardiac population. The unidimensional factor structure was confirmed through confirmatory factor analysis, accounting for 65.3% of total variance. Internal consistency was excellent, with Cronbach’s alpha of 0.89 and composite reliability of 0.90. Measurement invariance was established at scalar level across both age groups and gender, permitting meaningful score comparisons across demographic subgroups. These findings confirm GAD-7 suitability as a reliable anxiety screening instrument in coronary heart disease populations [214].

The Whooley questionnaire is a rapid, two-item yes/no depression screen that can be embedded into routine cardiology encounters because it is simple to administer and score. It asks whether, in the past month, patients have often been bothered by low mood and/or loss of interest/pleasure; any “yes” is considered a positive screen and should trigger follow-up diagnostic assessment and a defined care pathway. It is recommended by the National Institute for Health and Care Excellence for use in several clinical populations (e.g., chronic physical illness, prior depression, and perinatal care), supporting its pragmatic role as a first-step screen. Evidence from cardiovascular cohorts shows depressive symptoms predict adverse outcomes, reinforcing the value of systematic detection and linkage to intervention [80,215].

A diagnostic meta-analysis of ten studies established that the two-item Whooley questions, enquiring about depressed mood and anhedonia over the preceding month, demonstrated pooled sensitivity of 0.95 (95% CI: 0.88–0.97) and pooled specificity of 0.65 (95% CI: 0.56–0.74), with low between-study heterogeneity (I^2^ = 24.1%). The strongly negative likelihood ratio of 0.07 confirms their utility as a rule-out instrument: patients responding negatively to both items are highly unlikely to meet diagnostic criteria for major depressive disorder. However, modest specificity implies that a substantial proportion of positive screens will not fulfil diagnostic criteria, necessitating confirmatory clinical assessment following any positive result [216].

In high-throughput ACS units, the Whooley questions may serve as an initial rule-out step, followed by PHQ-9 administration in patients screening positive, thereby combining feasibility with improved specificity.

The Beck Depression Inventory-II (BDI-II) has been validated most extensively in post-ACS populations. A meta-analysis of four studies (*n* = 1576 post-ACS patients) estimated BDI-II sensitivity at 90% and specificity at 80% for major depressive disorder during hospitalization, confirming that depression screening can be performed with reasonable accuracy in this acute setting [217]. For anxiety, no single instrument has achieved consensus endorsement in ACS-specific guidelines.

However, diagnostic accuracy considerations are essential when selecting depression screening tools in post-ACS populations. Six observational studies (*n* = 1755) evaluated four instruments—BDI-II, GDS, HADS, and PHQ—predominantly in inpatient settings with low major depressive disorder prevalence. Across tools, sensitivity and specificity were generally acceptable, but positive predictive values were low, reinforcing the need for confirmatory clinical assessment after a positive screen. The BDI-II demonstrated sensitivity of 90% and specificity of 80%, with the most supporting data but longer administration time (5–10 min). HADS showed similar performance (sensitivity ~82%, specificity ~79%). PHQ-2 and PHQ-9 exhibited high sensitivity (~96%) but lower specificity (~71–72%). The GDS showed sensitivity of 100% and specificity of 83%, indicating slightly higher specificity than BDI-II in older adults [218].

#### 5.3.2. Diagnostic Challenges at the ACS–Mental Health Interface

Panic attacks, core features of panic disorder, can present with prominent cardiopulmonary symptoms (e.g., chest pain, palpitations, and dyspnea/breathlessness) [219].

Panic attacks can closely replicate ACS symptoms—chest tightness/discomfort, palpitations, dyspnea, diaphoresis, nausea/vomiting—making the two clinically indistinguishable without objective testing. The first panic attack is often spontaneous (DSM-IV-TR), further blurring bedside differentiation. Both conditions share a downstream pathway of sympathetic activation, producing similar autonomic and cardiopulmonary features. Because ACS cannot be excluded by history alone, evaluation should rely on ECG and cardiac biomarkers (troponin I preferred; CK-MB supportive), as panic attacks do not explain ischemic ECG changes or enzyme elevation; a normal ECG, however, does not rule out ACS [220].

A particularly challenging domain within this overlap is the evaluation of palpitations. In the differential diagnosis between medical events and panic attacks, palpitations represent a major overlap, as they may reflect true arrhythmias or benign cardiac sensations. Guidelines require physical examination, ECG, and detailed history for all patients with palpitations, since symptom perception alone is unreliable. Many individuals with confirmed arrhythmias are minimally symptomatic, whereas panic patients frequently report irregular heart rhythms without objective ECG abnormalities. Benign palpitations are strongly associated with psychiatric morbidity, including panic disorder, and interoceptive bias may amplify cardiac symptom reporting, complicating distinction from genuine cardiac pathology [221].

Clinically, this diagnostic convergence has measurable consequences. It has been observed that >20% of individuals seeking emergency cardiac evaluation may ultimately have panic disorder rather than an acute cardiac condition, often after negative cardiac testing. Thus, while premature reassurance risks overlooking ACS, premature psychiatric attribution may delay recognition of true ischemia, underscoring the need for structured, biomarker-supported assessment pathways [219].

Diagnostic overshadowing refers to the misattribution of physical symptoms to a pre-existing mental illness, resulting in underdiagnosis and inadequate treatment of somatic disease. It is driven by provider bias, stigma, anchoring, premature closure, and chart “flagging,” which predispose clinicians to interpret new complaints through a psychiatric lens. Patients may also internalize doubt about the legitimacy of their symptoms. Consequences include delayed investigation, worsening physical illness, reduced care-seeking, and potentially death. Recognition of this bias is essential to prevent compromised care and excess mortality among individuals with mental illness [222].

Moreover, this bias has measurable implications in acute cardiovascular care. In a retrospective emergency department cohort analysis (2009–2017), 1.3% of acute myocardial infarctions hospitalizations were preceded by a treat-and-release emergency department visit for nonspecific chest pain or dyspnea within the prior 30 days, classified as a probable “missed acute myocardial infarctions.” In this retrospective analysis, a history of MHD was associated with higher odds of missed acute myocardial infarctions (OR 1.48, 95% CI 1.23–1.77), with the highest risk in patients with co-occurring mental health and substance use disorders (OR 1.90, 95% CI 1.30–2.76). Substance use disorders alone were not significantly associated with missed the myocardial infarctions [223].

Beyond missed diagnoses, treatment disparities further support the presence of diagnostic overshadowing. Patients with mental illness and ischemic heart disease are less likely to receive revascularization, and those with comorbid diabetes are less often hospitalized for complications. This pattern appears mainly in individuals with non-psychotic disorders, while patients with psychosis do not show the same reduced admission rates [224].

In post-acute myocardial infarction (AMI) patients, common post-AMI symptoms (e.g., fatigue, reduced energy, and sleep and appetite disturbances) may be misclassified as depressive somatic symptoms, particularly when assessed using self-report instruments such as the BDI–II. In a study comparing post-AMI inpatients with matched psychiatric outpatients and students, somatic items (BDI–II items 15–21) accounted for a substantial proportion of total scores, especially at low overall depression levels (≈70% when BDI–II <4). However, post-AMI patients did not demonstrate clinically meaningful inflation of somatic scores compared with psychiatric outpatients matched on cognitive/affective symptoms and scored only approximately one point higher than matched students. These findings suggest minimal systematic overestimation, although a degree of residual somatic variance likely reflects illness-related symptom burden rather than depressive psychopathology [225].

Clinically, this overlap is important because somatic depressive symptoms may also reflect underlying cardiac status and prognosis. In ACS patients, somatic/affective symptoms (e.g., fatigue, insomnia, appetite or weight change, and functional impairment) overlap substantially with manifestations of acute cardiac illness, thereby increasing the risk of diagnostic misattribution. In a prospective cohort (*n* = 913; analyzed *n* = 874), only the somatic/affective dimension of depressive symptoms was associated with higher disease severity (Killip class: OR 1.49, 95% CI 1.23–1.81), whereas cognitive/affective symptoms were not. During 12-month follow-up (51 deaths), somatic/affective symptoms independently predicted all-cause mortality after adjustment (OR 1.92, 95% CI 1.36–2.71), while cognitive/affective symptoms showed no significant association. The authors emphasized that it remains uncertain whether these somatic symptoms represent depression per se or the physiological burden of ACS [226].

Changes in somatic symptoms following MI also appear prognostically relevant, further underscoring the clinical implications of attributing such symptoms solely to depression. In the ENRICHD secondary analysis, depressed post-MI patients (*n* = 1254) were assessed with the BDI at baseline and 6 months and followed for recurrent MI or all-cause mortality over approximately 2.4 years. Improvement in somatic symptoms was associated with better event-free survival (per 1-point improvement: HR 0.95, 95% CI 0.92–0.98), whereas change in cognitive symptoms was not (HR 0.98, 95% CI 0.96–1.01). After adjustment for demographic and clinical covariates, this association remained significant only in the CBT intervention arm (HR 0.93, 95% CI 0.88–0.98). These findings suggest that the trajectory of somatic symptoms carries independent prognostic information and should not be conceptualized as exclusively psychological in origin [227].

At the population level, longitudinal data from two large aging cohorts (HRS and ELSA; *n* = 17,787) demonstrated that depressive symptom trajectories over 8 years were associated with incident heart disease during 10-year follow-up. Persistently high (HR 1.64), increasing (HR 1.43), and fluctuating (HR 1.13) symptom patterns were linked to elevated cardiac risk, whereas decreasing symptoms were not. Notably, somatic depressive symptoms (e.g., fatigue, sleep disturbance, and low energy) exhibited stronger associations (persistently high HR 1.93) than cognitive-affective symptoms. Collectively, these findings indicate that somatic depressive features may both overlap with and signal underlying cardiovascular vulnerability, thereby creating a clinically significant risk of misattributing cardiac-related symptoms to primary depressive pathology [228].

Women with ACS present differently and experience worse outcomes than men, creating important diagnostic challenge. They are, on average, older at presentation, have a higher burden of comorbidities (e.g., diabetes, hypertension, and heart failure), and more frequently present with NSTE-ACS, NSTEMI, or MINOCA rather than STEMI. Women commonly report atypical symptoms such as dyspnea, fatigue, nausea, or back/neck pain, contributing to delayed recognition and treatment. Across multiple studies, women, particularly younger women, have higher in-hospital and 30-day mortality, more complications, and lower use of invasive procedures and guideline-based therapies. Psychosocial factors, including higher rates of depression and anxiety, further compound disparities in diagnosis, management, and recovery [229].

In the VIRGO study of young AMI patients (age 18–55; 2009 women and 976 men), chest pain/discomfort was the predominant symptom in both sexes (87.0% of women vs. 89.5% of men). However, women more often reported ≥3 additional non-chest symptoms (61.9% vs. 54.8%), such as epigastric complaints, palpitations, dyspnea, and jaw/neck/arm or interscapular pain. In adjusted analyses, women with STEMI were more likely than men to present without chest pain (OR 1.51, 95% CI 1.03–2.22). Women were more likely to attribute symptoms to stress/anxiety (20.9% vs. 11.8%) and presented later (median 3.2 vs. 2.4 h). Depressive symptoms (PHQ-9 ≥ 10) were more common in women (39.1% vs. 22.5%), and among those who sought care for similar symptoms before hospitalization, providers were more likely to dismiss a cardiac cause in women (53.4% vs. 36.7%) [230].

#### 5.3.3. Integration into Cardiology Workflow

Although both the American Heart Association and the U.S. Preventive Services Task Force formally support depression screening in cardiovascular populations, a comprehensive review of 65 cardiovascular clinical practice guidelines demonstrated limited incorporation of these recommendations into formal guidance documents. Only 37% of guidelines included explicit recommendations for depression screening, 34% addressed treatment strategies, and just 23% provided guidance on both screening and management. These findings underscore a substantial disconnect between high-level policy recommendations and their translation into detailed, operational clinical directives [231]. Accordingly, successful implementation requires embedding screening into predefined cardiology workflows rather than relying on discretionary provider-initiated assessment.

Translating screening recommendations into routine clinical practice within cardiology units requires deliberate structural adaptation of the care pathway rather than ad hoc provider-initiated assessment. Integration into electronic health records, through automated prompts at designated timepoints, conditional expansion from PHQ-2 to PHQ-9 when indicated, and clinical decision support alerts for positive screens, is a central strategy for achieving consistent, reproducible screening compliance [231,232].

In a single-center quality improvement depression screening protocol embedded in an AMI care pathway and administered by nursing staff, 26.8% of eligible patients were not screened. Concordance for positive screens between routine clinical screening and concurrent registry assessments was 35.6% for PHQ-2 and 61.5% for PHQ-9. Provider feedback suggested that simplifying the process and providing ongoing education/feedback could improve implementation [233]. These findings illustrate that screening accuracy and workflow simplicity are interdependent determinants of real-world compliance.

To facilitate integration into routine inpatient cardiac-unit workflow, the screening strategy leveraged an existing nursing admission data element as its initial step. Specifically, an iterative three-stage process was implemented in hospitalized cardiac patients: (1) a 4-item nurse-administered “Coping Screen” embedded in routine admission assessments; (2) for patients endorsing ≥1 Coping Screen item, a secondary 5-item screen administered by the study social work care manager that comprised the PHQ-2, the GAD-2, and a panic-attack question; and (3) for those with a positive 5-item screen, a diagnostic evaluation using the PHQ-9 and the PRIME-MD anxiety disorder modules to determine clinical depression, generalized anxiety disorder, or panic disorder. This approach demonstrated the feasibility of identifying both depression and anxiety disorders in an inpatient cardiology setting, with generalized anxiety disorder occurring at a prevalence similar to depression and with the GAD-2 functioning as an effective screening tool in this cohort [234].

#### 5.3.4. Proposed Post-ACS Screening Algorithm

**Figure 5:**
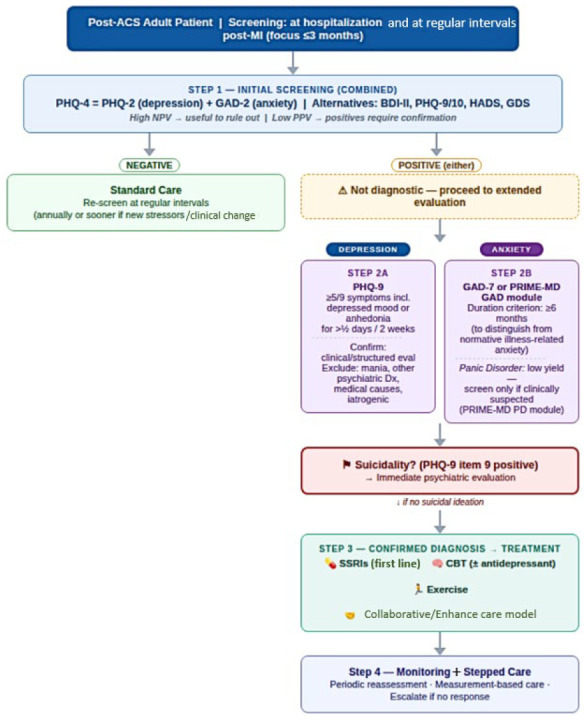
Structured algorithm for post-ACS mental health screening for depression and anxiety, incorporating initial combined screening, extended diagnostic evaluation, suicidality triage, and treatment with structured follow-up.

Based on the evidence reviewed, a structured stepwise algorithm for post-ACS mental health screening is proposed, integrating validated instruments for depression and anxiety, diagnostic confirmation criteria, and a treatment pathway within a collaborative care framework, as illustrated in Figure 5 [217,234,235]. However, mental health screening after ACS should be implemented only in clinical settings where robust systems are in place to ensure accurate diagnosis, evidence-based treatment, and structured longitudinal follow-up.

### 5.4. Cardiac Rehabilitation

Cardiac rehabilitation (CR) is designed to facilitate sustained functional recovery following major cardiovascular events, including ACS, and therefore constitutes a strategic platform for incorporating secondary prevention of adverse mental health outcomes. From this perspective, comprehensive CR models that integrate patient education, structured physical training, and psychosocial counseling have been shown to yield meaningful reductions in post-ACS depressive and anxiety symptoms, particularly among individuals presenting with elevated baseline psychological distress and demonstrating adequate program adherence [236,237].

Multimodal exercise-centered CR programs implemented after ACS combine aerobic conditioning, resistance modalities, and structured educational components and have repeatedly demonstrated favorable effects on cognitive performance, thereby reinforcing secondary prevention strategies and long-term engagement with care. Interventions encompass stretching routines, home-based or remotely supervised training, virtual reality-supported exercise, promotion of activities of daily living, breathing techniques, and sport-oriented therapy. Educational curricula typically focus on nutrition, smoking cessation, regular physical activity, stress reduction, medication adherence, management of coronary risk factors, and overall disease self-management, with some protocols also embedding psychological counseling and dietary guidance. In selected randomized trials, aerobic and resistance workloads were prescribed at approximately 65–70% of heart rate reserve. Collectively, these programs exert broad benefits across cognitive domains, cardiopulmonary performance, functional capacity, psychological well-being, and health-related quality of life in individuals recovering from ACS [238].

Mobile health platforms provide a practical and scalable avenue for delivering CR following hospital discharge. This concept was validated in the SMART-REHAB study, which demonstrated the feasibility and clinical utility of a smartphone-based early rehabilitation program for individuals recovering from ACS [239].

Contemporary American Academy of Family Physicians recommendations underscore the importance of coordinated strategies that address the two-way interaction between ACS and psychiatric morbidity. Systematic screening for depressive symptoms is advised within the first three months following an ACS episode, using standardized and validated instruments, with subsequent diagnostic confirmation when indicated, despite the underlying evidence being of low certainty. When depression is established in the post-ACS setting, the AAFP strongly supports initiation of antidepressant pharmacotherapy, preferably with SSRIs or SNRIs, and/or structured psychotherapeutic interventions such as CBT, based on moderate-quality evidence. Agents from the SSRI class are generally preferred to TCAs because of their more favorable cardiovascular safety profile. Models incorporating collaborative care and stepwise treatment escalation, integrating medication, psychotherapy, and organized follow-up, yield superior improvement in depressive symptoms compared with routine care alone. While robust reductions in major cardiovascular events have not been consistently demonstrated, effective management of depression is associated with better quality of life, greater treatment adherence, and enhanced functional recovery, justifying close integration with CR programs and multidisciplinary cardiovascular services [218].

Embedding structured psychological therapies into multidisciplinary CR, together with lifestyle optimization and standard medical care, yields measurable benefits across the psychiatric disorder–CVD continuum. Among patients with ACS, HF, and those recovering from cardiac surgery, three modalities—group-based psychoeducation, progressive muscle relaxation using Jacobson’s method combined with imaginative stabilization, and individualized counseling—produce significant reductions in anxiety and depressive symptoms. These interventions also enhance patients’ perceptions and understanding of their illness, with improvements evident at program completion and maintained at three-month follow-up [240].

### 5.5. Clinical Evidence on MHD-ACS Axis

The exploration of the clinical relevance of the MHD-ACS bidirectional axis must be supported by continuous research, particularly through well-designed clinical trials. A growing number of interventional and observational studies across various phases and designs underline the need to clarify whether targeted treatment of mental health disorders after ACS improves cardiovascular outcomes and whether cardiovascular-focused interventions influence psychiatric trajectories. This relevance is reflected by multiple trials registered in the ClinicalTrials.gov database evaluating pharmacological, psychotherapeutic, exercise-based, and collaborative care strategies in patients with ACS and comorbid mental health disorders. Therefore, a structured search was performed in the ClinicalTrials.gov database using “Acute Coronary Syndrome” in the Condition/disease field and “Mental Health” in the Other terms field, without specifying intervention type or geographic location. No restrictions were applied regarding study status in order to capture ongoing, completed, and terminated trials relevant to the ACS–mental health axis. The search strategy identified a total of 51 studies, of which 11 reported available results and were therefore selected for further evaluation. Table 3 presents a standardized framework for reporting clinical trials investigating the ACS–MH axis [241].

### 5.6. Clinical Translation Through Risk Prediction Models

Despite accumulating evidence that psychiatric comorbidities, particularly depression and anxiety, independently predict adverse outcomes following ACS, none of the currently employed acute cardiovascular risk stratification tools, including GRACE [242], TIMI, and HEART [243], incorporate mental health variables.

Relevant methodological precedents do exist at the interface of psychiatry and cardiovascular risk modeling. The QRISK3 algorithm, developed and validated by Hippisley-Cox et al. in a prospective open cohort of over 7.89 million patients, was the first major cardiovascular risk prediction tool to formally incorporate severe mental illness and atypical antipsychotic use as independent predictors of 10-year cardiovascular risk, demonstrating improved reclassification performance over its predecessor. This establishes proof of concept that psychiatric variables can meaningfully augment cardiovascular risk algorithms without compromising discriminatory accuracy [244].

However, the most recent iteration of this algorithmic lineage, QR4, derived and externally validated across 16.77 million adults, identified seven novel risk factor categories including chronic obstructive pulmonary disease, learning disability, and multiple cancer types, yet did not extend this framework to incorporate additional MHDs variables beyond those present in QRISK3. This highlights that the systematic integration of MHD-related predictors into cardiovascular risk algorithms remains an unresolved challenge, even within contemporary modeling efforts [245].

Nevertheless, no validated prognostic score specifically designed for the acute ACS setting, the context in which cardiologists and emergency physicians make time-sensitive triage and treatment decisions, integrates psychiatric variables. This represents a clinically meaningful translational gap. Prospective development and validation of augmented ACS risk prediction models that incorporate standardized psychiatric assessments constitute a high-priority research direction, with direct implications for post-discharge risk stratification, follow-up intensity, and multidisciplinary care pathways.

### 5.7. Systems-Level Disparities in ACS Care Among Patients with MHD

Beyond biological and behavioral mechanisms, patients with severe mental illness consistently experience disparities in the delivery of evidence-based cardiovascular care [246,247].

Data from a comparative meta-analysis indicate that individuals with MHDs have been reported to be less likely to receive timely reperfusion therapy during acute myocardial infarction, including primary percutaneous coronary intervention. Delays in presentation, diagnostic overshadowing, and reduced access to specialized cardiac centers have been proposed as contributing factors [26,248].

Medication adherence represents an additional critical barrier. Cognitive impairment, negative symptoms, socioeconomic instability, fragmented healthcare access, and polypharmacy contribute to lower adherence rates to cardiovascular medications in this population. Consequently, excess cardiovascular mortality in patients with MHDs likely reflects not only pathophysiological vulnerability but also structural and systems-level inequities in care delivery [249,250].

Addressing the MHD–ACS interface therefore requires not only pharmacologic and psychotherapeutic optimization but also targeted strategies to improve equitable access to reperfusion therapy, adherence to guideline-directed medical therapy, and continuity of cardiovascular follow-up.

## 6. Conclusions and Prospects

MHDs and ACSs constitute a bidirectional continuum characterized by shared inflammatory, neuroendocrine, autonomic, and behavioral pathways. Evidence across epidemiological, mechanistic, and clinical studies consistently supports this interdependence across diverse populations and clinical settings. Depression, anxiety, PTSD, and severe mental illness significantly worsen cardiovascular prognosis, yet systematic screening and integrated care remain underutilized despite demonstrated efficacy.

Future research priorities include: (1) randomized trials evaluating whether treating post-ACS psychiatric disorders reduces major adverse cardiac events and mortality; (2) inflammatory and genetic biomarkers enabling risk stratification and personalized intervention; (3) implementation studies addressing barriers to routine mental health screening in cardiology; (4) incorporation of cardio-psychiatric collaboration into clinical guidelines and healthcare systems; and (5) novel psychopharmacological and neuromodulatory therapies for cardio-psychiatric populations. Integrating mental health assessment into cardiovascular pathways represents an urgent priority for improving outcomes.

## Figures and Tables

**Table 1:** Mental health phenotypes across the ACS continuum.

Mental Health Phenotype	Prevalence in ACS Populations	Directionality with ACS	Prognostic/Clinical Impact in ACS	Ref.
Severe mental illness (overall)	~4.1% of ACS patients	Bidirectional	Higher short- and long-term mortality; lower revascularization and cardioprotective pharmacotherapy	[27]
Severe mental illness (STEMI, PCI treated)	~3.8%	ACS → worse outcomes	Higher MACEs over follow-up (HR 1.27–1.43), mainly driven by mortality	[25]
Schizophrenia	Not specified	Bidirectional	≈70% higher mortality after ACS; higher MACEs, stroke, and bleeding; lower revascularization and cardioprotective therapy	[28]
Psychological distress (any)	17.6%	ACS → distress	Common post-ACS morbidity phenotype	[29]
Depression	15.6–31.3%; ~20% major depressive disorder	Bidirectional	1.59–2.71× higher risk of mortality or new CV events after MI	[37]
Anxiety	20–50% after MI	Bidirectional	Baseline anxiety associated with 21% higher mortality and 47% higher MACEs	[44]
PTSD (ACS induced)	~12% (up to ~16% by questionnaires)	Bidirectional	≈2-fold higher risk of recurrent cardiac events and/or mortality	[53]
Bipolar disorder	~1.3% of ACS hospitalizations	Bidirectional	Higher MACEs, all-cause mortality, and stroke after ACS	[63]
Suicidal ideation	14.1%	ACS → SI (predominant)	45% higher SI risk vs. controls; highest within first 6 months	[68]
Suicide (completed)	Not prevalence based	ACS → suicide	aOR 1.15; OR 3.05 in first 6 months post-ACS	[70]
Genetic susceptibility to suicidal ideation	20% within 2 weeks; 12% at 1 year	ACS → SI (acute phase)	5-HTTLPR s allele associated with SI only in acute phase	[71]

ACS, acute coronary syndrome; MACE, major adverse cardiovascular event; MI, myocardial infarction; PCI, percutaneous coronary intervention; PTSD, post-traumatic stress disorder; SI, suicidal ideation; HR, hazard ratio; OR, odds ratio; aOR, adjusted odds ratio. Directionality indicates whether evidence supports mental health disorders as risk factors for incident coronary disease and/or as consequences of ACS. Prevalence estimates reflect ranges reported across large observational cohorts and meta-analyses. Prognostic impact refers to associations with mortality, recurrent ischemic events, stroke, or adverse clinical outcomes after ACS.

**Table 2:** Mechanistic pathways linking MHD-ACS.

Mechanistic Domain	Mental Health Disorders → Biological Alterations	Shared Mediators	Cardiovascular Consequences → ACS	Ref.
Chronic low-grade inflammation and immune activation	Psychiatric disorders show systemic and central pro-inflammatory profile with increased IL-1β, IL-2, IL-6, IL-8, IL-12, IFN-α, CCL2, TNF-α, CRP, CXCL4, CXCL7, IBA1, TLR3, and TLR4 and reduced IL-4, CCL4, TGF-β, and BDNF	IL-1β, IL-6, TNF-α, CRP, NF-κB, NLRP3, JAK/STAT	Macrophage-driven inflammation promotes plaque formation; amplified cytokine signaling contributes to cap thinning and destabilization; IL-6 associated with cardiovascular death, MI, stroke, PAD, and HF	[118,122,123,125]
Autonomic nervous system dysregulation	Chronic stress induces sympathetic predominance and vagal withdrawal via CRH signaling; sustained norepinephrine and epinephrine elevations with reduced acetylcholine	CRH, norepinephrine, epinephrine	Sympathetic overactivity promotes endothelial dysfunction, arterial stiffness, LV hypertrophy, RAAS activation, adverse remodeling, and predisposition to ACS	[151,155,156]
HPA axis hyperactivation	Increased CRH/AVP drive ACTH and sustained cortisol release; impaired GR-mediated feedback; disrupted circadian cortisol; reduced BDNF; altered serotonergic signaling; hippocampal and PFC damage	CRH, AVP, ACTH, cortisol, GR	Higher cortisol associated with increased cardiovascular risk; endothelial glucocorticoid resistance permits persistent cytokine signaling and NO suppression	[160,161,163,171]
Platelet hyperreactivity and prothrombotic state	Altered platelet serotonergic/noradrenergic signaling in depression; morphological and metabolic abnormalities in schizophrenia; oxidative stress alters ADP/collagen responses; reduced platelet benzodiazepine receptor density in anxiety/suicidality	Serotonin, ADP, thrombo-inflammatory signaling	Plaque rupture exposes collagen and vWF → platelet adhesion via GPIb-IX-V, GPVI, and α2β1 → platelet accumulation, hyperreactivity, occlusive thrombus, and myocardial ischemia	[165,166,168]
Endothelial dysfunction	Impaired endothelium-dependent vasodilation due to reduced NO bioavailability; increased cytokines and acute-phase proteins disrupt endothelial signaling; attenuated HDL protective effects	↓NO, cytokines, CRP, cortisol	Endothelial dysfunction independently predicts mortality in ACS (~2.4-fold higher cardiovascular death risk)	[169,170,171]

ACTH, adrenocorticotropic hormone; ACS, acute coronary syndrome; ADP, adenosine diphosphate; AVP, arginine vasopressin; BDNF, brain-derived neurotrophic factor; CAD, coronary artery disease; CRH, corticotropin-releasing hormone; CRP, C-reactive protein; GR, glucocorticoid receptor; HDL, high-density lipoprotein; HF, heart failure; IFN-α, interferon alpha; IL, interleukin; JAK/STAT, Janus kinase/signal transducer and activator of transcription; LV, left ventricular; MI, myocardial infarction; NF-κB, nuclear factor kappa B; NO, nitric oxide; PAD, peripheral artery disease; PFC, prefrontal cortex; RAAS, renin–angiotensin–aldosterone system; TGF-β, transforming growth factor beta; TLR, toll-like receptor; TNF-α, tumor necrosis factor alpha; vWF, von Willebrand factor; ↓ decreased levels or activity.

**Table 3:** Clinical trials investigating the MHD-ACS axis.

Database ID	Official Title	Description	Key Outcomes
NCT03122184	Positive Psychology for Acute Coronary Syndrome Patients (PEACE-IV)	Randomized, single-blind, parallel-group pilot RCT in ACS patients (*n* = 69); twelve-week telephone-delivered behavioral intervention comparing positive psychology + motivational interviewing versus motivational interviewing health education	PP + MI was feasible, well accepted, and produced greater improvements in physical activity, positive affect, optimism, and overall mental well-being than motivational interviewing health education
NCT02004158	Positive Psychology to Improve Healthy Behaviors After an Acute Coronary Syndrome (PEACE II)	Open-label, single-arm proof-of-concept interventional study assessing feasibility, ease, and psychological impact of a positive psychology program using self-report and clinician-rated scales	Positive psychology exercises were feasible, easy to complete, increased optimism and positive affect, and reduced anxiety/depressive symptoms, supporting potential as adjunctive therapy after ACS
NCT01032018	Comparison of Depression Interventions After Acute Coronary Syndrome (CODIACS)	Randomized, single-blind, parallel-group interventional trial comparing stepped-care depression management versus referred/usual care in post-ACS patients, using BDI-based mixed-model analyses	Stepped-care produced greater reduction in depressive symptoms than referred care, with similar healthcare utilization and no safety concerns, supporting stepped-care for post-ACS depression
NCT01993017	Comparison of Depression Identification After Acute Coronary Syndrome: Quality of Life and Cost Outcomes (CODIACSQoL)	Large multicenter randomized, single-blind, parallel-group screening trial comparing AHA screen-and-treat, screen-and-notify, and no-screen strategies; outcomes analyzed using ANOVA and superiority testing	Depression screening with or without stepped-care treatment did not improve quality-adjusted life years, depression-free days, or costs versus no screening in post-ACS patients
NCT00998400	Treatment of Depression in Acute Coronary Syndrome (ACS) Patients (TREATED-ACS)	Open-label, randomized, parallel-group interventional trial in post-ACS patients with DSM-IV depressive disorders and/or DCPR demoralization; 12 weekly psychotherapy sessions; repeated-measures ANOVA with ITT and multiple imputation	CBT + well-being therapy + lifestyle modification improved clinician-rated depressive symptom severity versus clinical management; hostility symptoms also improved; no consistent between-group benefits across anxiety, self-reported depression/somatization, or well-being dimensions
NCT00822679	Eszopiclone and Inflammatory Mediators in Patients with Acute Coronary Syndrome	Randomized, double-blind, placebo-controlled phase 4 interventional trial; parallel assignment; planned biomarker and sleep outcome analysis, but no statistical analysis performed due to zero randomized participants	No efficacy or safety outcomes available; study terminated without data because no participants were successfully randomized or analyzed
NCT05328375	Telehealth-enhanced Hybrid Cardiac Rehabilitation Among Acute Coronary Syndrome Survivors	Pilot randomized, open-label, parallel-group feasibility RCT comparing telehealth-enhanced hybrid cardiac rehabilitation versus traditional cardiac rehabilitation; descriptive and pre–post within-group analyses of adherence and functional outcomes	THCR demonstrated high feasibility and strong session adherence, universal program initiation in both arms, and meaningful pre–post improvements in functional capacity and quality of life, supporting scalability of hybrid CR after ACS
NCT04983680	Remote-delivered MBCT for SCAD Survivors	Interventional; open-label, single-group pilot (feasibility/acceptability); pre–post self-report surveys + actigraphy; exploratory pre–post change analyses and inter-correlation of psychological/behavioral outcomes.	Remote MBCT was feasible/acceptable with high enrollment/retention and good session attendance/home practice; fear of recurrence and cardiac anxiety decreased; cognitive decentering increased; interoceptive bias and intolerance of uncertainty decreased; sleep disturbances slightly improved and self-reported physical activity increased
NCT05299723	The SleepWell Study—Chronotherapeutic Intervention to Improve Sleep Following ACS	Two-phase interventional pilot: Phase A single-arm open-label combined chronotherapy; Phase B randomized parallel pilot vs. sleep hygiene control; assessments: feasibility/acceptability/usability questionnaires, adherence logs, and pre–post sleep questionnaires	Intervention generally feasible and usable; adherence acceptable; sleep symptoms improved versus baseline, with greater improvements in combined chronotherapy than sleep hygiene control across insomnia severity, sleep quality, and sleep duration
NCT03605693	Early Psychological Intervention to Prevent Cardiovascular Event-Induced PTSD (REACH Sub-study)	Pilot randomized parallel trial comparing written exposure therapy versus usual care; blinded assessor PTSD evaluation; feasibility, adherence, depression, and medication adherence self-report measures	Written exposure therapy feasible and well accepted; PTSD and depressive symptoms comparable or lower than usual care; no clear medication adherence difference observed
NCT01566214	Vet-Harts Pilot Intervention for Veterans with Coronary Heart Disease (VHPI)	Pilot randomized parallel trial evaluating a telehealth motivational interviewing nursing intervention versus usual care, using patient-reported quality-of-life and angina questionnaires	Usual care showed greater improvements in physical, emotional, and role functioning; motivational interviewing showed modest benefits mainly in disease perception and angina stability.

PP, positive psychology; MI, motivational interviewing; ACS, acute coronary syndrome; CBT, cognitive behavioral therapy; DSM-IV, Diagnostic and Statistical Manual of Mental Disorders, fourth edition; DCPR, Diagnostic Criteria for Psychosomatic Research; ANOVA, analysis of variance; ITT, intention to treat; CR, cardiac rehabilitation; THCR, telehealth-enhanced hybrid cardiac rehabilitation; RCT, randomized controlled trial; MBCT, mindfulness-based cognitive therapy; PTSD, post-traumatic stress disorder.

## Data Availability

No new data were created or analyzed in this study.

## Abbreviations

The following abbreviations are used in this manuscript:

ACS	Acute Coronary Syndrome
CAD	Coronary Artery Disease
CVD	Cardiovascular Disease
ASCVD	Atherosclerotic Cardiovascular Disease
MHD	Mental Health Disorder
PTSD	Post-Traumatic Stress Disorder
MI	Myocardial Infarction
NSTEMI	Non-ST Segment Elevation Myocardial Infarction
STEMI	ST Segment Elevation Myocardial Infarction
NSTE-ACS	Non-ST Segment Elevation Acute Coronary Syndrome
MACE	Major Adverse Cardiac Event
HF	Heart Failure
PCI	Percutaneous Coronary Intervention
ECG	Electrocardiogram
HPA	Hypothalamic–Pituitary–Adrenal (axis)
CRH	Corticotropin-Releasing Hormone
AVP	Arginine Vasopressin
ACTH	Adrenocorticotropic Hormone
NO	Nitric Oxide
CBT	Cognitive Behavioral Therapy
SSRI	Selective Serotonin Reuptake Inhibitor
SNRI	Serotonin–Norepinephrine Reuptake Inhibitor
TCA	Tricyclic Antidepressant
MAOI	Monoamine Oxidase Inhibitor
CR	Cardiac Rehabilitation
RCT	Randomized Controlled Trial
MBCT	Mindfulness-Based Cognitive Therapy
DSM-IV	Diagnostic and Statistical Manual of Mental Disorders, Fourth Edition
ITT	Intention to Treat
ADP	Adenosine Diphosphate
vWF	von Willebrand Factor
BDNF	Brain-Derived Neurotrophic Factor
IL	Interleukin
TNF-α	Tumor Necrosis Factor Alpha
CRP	C-Reactive Protein
MR	Mendelian Randomization
GWAS	Genome-Wide Association Study
CAC	Coronary Artery Calcification
HRV	Heart Rate Variability
PHQ-9	Patient Health Questionnaire-9
HADS-D	Hospital Anxiety and Depression Scale–Depression Subscale
DSM	Diagnostic And Statistical Manual of Mental Disorders
GAD-7	Generalized Anxiety Disorder-7
BDI-II	Beck Depression Inventory-II
